# Significant role of symbiotic bacteria in the blood digestion and reproduction of *Dermanyssus gallinae* mites

**DOI:** 10.1093/ismeco/ycae127

**Published:** 2024-10-30

**Authors:** Qi Liu, Tiancong Sun, Penglong Wang, Lifang Wang, Helena Frantova, David Hartmann, Jan Perner, Weiwei Sun, Baoliang Pan

**Affiliations:** National Key Laboratory of Veterinary Public Health Security, College of Veterinary Medicine, China Agricultural University, Hai Dian District, Beijing 100193, China; National Key Laboratory of Veterinary Public Health Security, College of Veterinary Medicine, China Agricultural University, Hai Dian District, Beijing 100193, China; National Key Laboratory of Veterinary Public Health Security, College of Veterinary Medicine, China Agricultural University, Hai Dian District, Beijing 100193, China; National Key Laboratory of Veterinary Public Health Security, College of Veterinary Medicine, China Agricultural University, Hai Dian District, Beijing 100193, China; Institute of Parasitology, Biology Centre of the Czech Academy of Sciences, České Budějovice, Czech Republic; Institute of Parasitology, Biology Centre of the Czech Academy of Sciences, České Budějovice, Czech Republic; Institute of Parasitology, Biology Centre of the Czech Academy of Sciences, České Budějovice, Czech Republic; National Key Laboratory of Veterinary Public Health Security, College of Veterinary Medicine, China Agricultural University, Hai Dian District, Beijing 100193, China; National Key Laboratory of Veterinary Public Health Security, College of Veterinary Medicine, China Agricultural University, Hai Dian District, Beijing 100193, China

**Keywords:** poultry red mite, symbiotic bacteria, *Bartonella* A, antibiotic treatment, blood digestion, reproduction

## Abstract

Endosymbiotic bacteria significantly impact the fitness of their arthropod hosts. *Dermanyssus gallinae*, the poultry red mite, is a blood-feeding ectoparasite that exclusively feeds on avian blood. While there is a relatively comprehensive understanding of its microbial community structures across developmental stages based on 16S rRNA sequencing， the functional integration of these microbes within the host’s physiology remains elusive. This study aims to elucidate the role of symbiotic bacteria in *D. gallinae* biology. 16S rRNA amplicon sequencing and fluorescence *in situ* hybridization revealed a prominent midgut-confinement bacterial microbiota with considerable diversity, out of which *Kocuria* and *Bartonella* A acted as the predominant bacterial genera inhabiting *D. gallinae*. The relative abundance of *Bartonella* A increased rapidly after blood-sucking, suggesting its adaptation to a blood-based diet and its pivotal role in post-engorgement activities. Some of the isolated bacterial strains from *D. gallinae* display hemolytic activity on blood agar, potentially aiding blood digestion. To corroborate this *in vivo*, antibiotic-mediated clearance was exploited to generate dysbiosed cohorts of *D. gallinae* mites, lacking some of the key bacterial species. Phenotypic assessments revealed that dysbiosed mites experienced delayed blood digestion and diminished reproductive capacity. Whole-genome sequencing identified *Bartonella* A as a new species within the genus *Bartonella*, exhibiting characteristics of an obligate symbiont. These findings underscore the significance of microbiota in poultry red mites and suggest microbiota-targeted strategies for controlling mite populations in poultry farms.

## Graphical Abstract

**Graphical Abstract:**
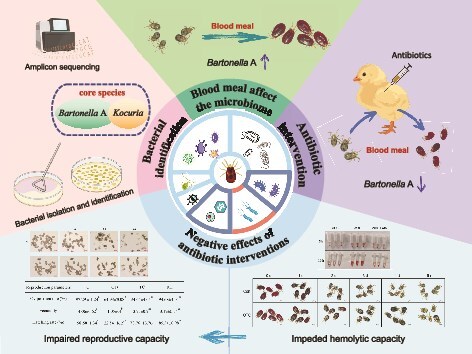

## Introduction

*Dermanyssus gallinae*, the poultry red mite, is a notorious hematophagous ectoparasite infesting poultry and wild birds, with a particular emphasis on laying hens on a global scale [1]. Infestations by *D. gallinae* can lead to severe detrimental effects, such as anemia and malnutrition, resulting in reduced egg production, compromised egg quality, increased morbidity, and even mortality of birds [2]. In recent years, the ubiquitous presence and profound impact of *D. gallinae* have drawn significant attention not only for their direct harm to avian hosts but also for their potential role as vectors in the transmission of pathogens, highlighting the critical intersection of animal, human, and environmental health within the One Health framework. Practically, the infestation of *D. gallinae* imposes huge economic losses on the poultry industry, estimated at €231 million annually in Europe alone [1, 3–7].

Microbial populations are prevalent within arthropods, exhibiting a wide range of effects on fitness of their hosts, such as providing protection against parasites and microbial pathogens, facilitating the degradation of chemical pesticides [8], supplying essential nutrients [9], and stimulating immune responses (see comprehensive reviews for details: [10, 11]). Blood-feeding arthropods, such as ticks [12] and mosquitoes [13], also exhibit a complex relationship with their bacterial microbiota. Symbiotic bacteria play an important role in their physiology, including blood digestion, synthesis of nutrients, and regulation of immunity [14]. Understanding the intricate structure and function of these microbial communities is essential for elucidating the mechanisms underlying the relationship between blood-feeding arthropods and their symbionts, and for informing strategies aimed at mitigating the public health risks posed by these vectors. *Dermanyssus gallinae* is a strict blood-feeder. A relatively comprehensive understanding of their microbial community structures has recently been achieved, mainly based on 16S rRNA gene amplicon sequencing [4, 15]. A considerable diversity of microorganisms has been discovered within *D. gallinae* with *Bartonella* A bacteria constituting the most dominant group within the *D. gallinae* microbiome, as they were detected across all developmental stages and sampling sites [4]. A recent study conducted on the microbiome of *D. gallinae* in a Japanese population also demonstrated the presence of *Bartonella* sp. group A in all individual mites, suggesting possible vertical transmission through eggs and the potential significance of *Bartonella* in mite survival [16]. Current studies on the symbiotic bacteria of *D. gallinae* mites are mainly based on PCR amplification and 16S rRNA gene amplicon sequencing methods, and to date, no isolated bacteria are available. The absence of pure cultures poses a challenge in accurately determining microbial features, including growth characteristics, metabolism, physiology, and cell biology for individual organism and attributes that are not easily inferred from genome sequences alone. Therefore, gaining insights into the characteristics of culturable bacteria becomes imperative.

The culturability of the *D. gallinae* microbiota together with the antibiotic-mediated dysbiosis model of mites serve as key groundwork for investigating the functional roles of these symbiotic bacteria. In the present study, several antibiotics were used to intervene with the bacteria in *D. gallinae*, and the phenotypic changes in the mites were observed after antibiotic treatments, with the goal of revealing the intricate relationship between symbiotic bacteria and the physiological aspects of the mite hosts. Specific attention was paid to blood digestion and following reproductive capacity of female mites, as these two parameters largely determine the fitness of blood-sucking arthropods and thus the impact on mite populations on farms and well-being of farmed hens. Therefore, understanding the role of symbiotic bacteria in blood digestion and reproduction of *D. gallinae* represents a promising avenue for future research and potential control strategies for managing populations of this pest.

## Materials and methods

### Birds and *D. gallinae* colonies

All chicks (Jingbai 939 strain, females) used in this study were obtained from a commercial hatchery on day 1 and were kept in metal cages and plastic storage boxes under laboratory conditions, with free access to feed and water until use. The storage boxes with chicks were placed in an artificial climate incubator (RXZ-500B-LED; Ningbo Jiangnan Instrument Factory, Ningbo, China) at 30°C and 75% relative humidity (RH), with a 12/12 h light/dark photoperiod [17].

The *D. gallinae* colony used in the present study was originally obtained from a commercial poultry farm in China, which was reared in the *in vivo* system at 30°C, 75% RH under laboratory conditions for ~5 years [17].

### Fluorescence *in situ* hybridization

To localize the bacteria within the mite, whole-mount fluorescence *in situ* hybridization (FISH) targeting bacterial 16S rRNA was performed. A universal 16S rRNA bacterial probe (5′-GCTGCCTCCCGTAGGAGT-3′) labeled with Cy5 at the 5′ end was used for FISH. A sense probe (5′-ACTCCTACGGAGGCAGC-3′) served as a control. These probes were synthesized by Sangon Biotech (Shanghai, China). FISH of eggs, larvae, nymphs, and adults was performed following the previously described method [18] with minor modifications. First, the specimens were placed in a 20-fold volume of Carnoy’s fixative (chloroform/ethanol/glacial acetic acid, 6:3:1) and fixed overnight at room temperature. After fixation, they were rinsed in 1 mL of 100% ethanol for 5 min, three times. Then, they were bleached with 6% hydrogen peroxide in ethanol at room temperature. The bleaching time was determined based on the color of the mite’s body. When the color became white, it indicated that the decoloration was completed. It took 6 h for eggs and larvae, 12 h for nymph, and 24 h for adults for bleaching. The bleached samples were washed three times with 1 mL of 70% ethanol to remove H_2_O_2_, and then the samples were hydrated by incubating with PBSTx buffer (phosphate buffer with 0.3% Triton X-100) three times. The samples were pre-incubated with 1 mL of probe-free hybridization buffer (20 mM Tris–HCl [pH 8.0], 0.9 M NaCl, 0.01% sodium dodecyl sulfate, 30% formamide) three times at room temperature by shaking (20 rpm) for 5 min each time, and then incubated with the hybridization buffer containing the probes (10 pmol/mL) overnight at 45°C with shaking (200 rpm) in darkness. After removing the hybridization buffer, 500 μL of the prewarmed washing buffer (20 mM Tris HCl [pH 8.0], 80 mM NaCl, 50 mM ethylenediaminetetraacetic acid, and 0.01% SDS) was added. Subsequently, 1 mL of fresh prewarmed washing buffer was introduced, and the samples were incubated for another 30 min at 45°C. The washing buffer was then changed twice with sterile water. Finally, the stained samples were transferred to sterilized slides; after absorbing excess water with filter paper, 50% glycerol (PBS/glycerol, 1:1) was added dropwise to the samples, covered with coverslips, and fixed with Fixogum around the perimeter. The samples were viewed under a Nikon A1HD25 confocal microscope and the fluorochrome Cy5 was detected using a 640-nm laser line and a BA 660IF filter.

### Mite collection and DNA extraction for amplicon sequencing

Eggs (E), fed adult females (AF), and adult males (AM) were collected from the rearing system. Additionally, to examine changes in bacterial composition after a blood meal, engorged adult female mites (EA) and starved adult female mites (SA) were compared. SA were obtained by incubating EA in an artificial climate incubator at 37°C and 75% RH for 5 days. Three replicates were set up for each sample used for amplicon sequencing, and the samples were named E1, E2, E3, AF1, AF2, AF3, AM1, AM2, AM3, EA1, EA2, EA3, SA1, SA2, and SA3, respectively. Since the AF and EA groups have the same feature, they were tested only once in this study, but they were denoted as different names, e.g. AF (in the analysis of the structure of the microbiota in *D. gallinae* across stages) or EA (in the analysis of the structure of the microbiota in *D. gallinae* affected by blood meal), to make it seem relative to the partner name. Samples of mites (100 individuals per sample) or eggs (200 eggs per sample) were surface-cleaned following the protocol described in previous studies [4] with minor modifications. In brief, the samples were washed three times with phosphate-buffered saline with Tween 20 (PBST) for 5 min each time, followed by the addition of 75% ethanol and soaking for more than 3 h. Subsequently, the samples underwent three washes with sterile water, and bacteria DNA was amplified from the last water after washing of mites/eggs to verify the efficacy of surface cleaning (Supplementary Fig. S1). The surface-cleaned samples were then homogenized for genomic DNA extraction. Microbial genomic DNA was extracted from the homogenate using the DNeasy Blood & Tissue Kit (Qiagen, Hilden, Germany) according to the manufacturer’s instructions.

### Amplicon sequencing

The microbial population of each sample were then analyzed separately using 16S rRNA amplicon sequencing by Shanghai Majorbio Bio-pharm Technology Company Limited (Shanghai, China). Briefly, the hypervariable region V3–V4 (~464 bp) of the bacterial 16S rRNA gene was amplified using primer pairs 338F and 806R (Supplementary Table S1) by an ABI GeneAmp® 9700 PCR thermocycler (ABI, CA, USA). The PCR amplification of the 16S rRNA gene was performed as follows: initial denaturation at 95°C for 3 min, followed by 30 cycles of denaturing at 95°C for 30 s, annealing at 55°C for 30 s and extension at 72°C for 45 s, and a single extension at 72°C for 10 min, and ending at 10°C. The PCR mixtures contained 5× TransStart FastPfu buffer (4 μL), 2.5 mM dNTPs (2 μL), forward primer (5 μM) (0.8 μL), reverse primer (5 μM) (0.8 μL), TransStart FastPfu DNA Polymerase (0.4 μL), template DNA (10 ng), and finally ddH_2_O up to 20 μL. PCR reactions were performed in triplicate. PCR amplifications were performed in quintuplicate for each sample. The PCR product was extracted from a 2% agarose gel, purified using the AxyPrep DNA Gel Extraction Kit (Axygen Biosciences, Union City, CA, USA) according to the manufacturer’s instructions, and quantified using a Quantus™ Fluorometer (Promega, USA). Purified amplicons were pooled equimolarly and paired-end sequenced on an Illumina MiSeq PE300 platform (Illumina, San Diego, USA) according to the standard protocols by Majorbio Bio-Pharm Technology Co. Ltd. (Shanghai, China).

### Bioinformatic analysis of retrieved 16S amplicon sequences

Raw fastq files were demultiplexed, quality-filtered using fastp version 0.20.0 [19], and merged by FLASH version 1.2.7 [20] with the following criteria: (i) the PE300 reads were truncated at any site receiving an average quality score of <20 over a 50-bp sliding window, and the truncated reads shorter than 50 bp were discarded; reads containing ambiguous characters were also discarded. (ii) Only overlapping sequences longer than 10 bp were assembled based on their overlapped sequence. The maximum mismatch ratio of the overlap region was 0.2. Reads that could not be assembled were discarded. (iii) Samples were distinguished based on the barcode and primers, and the sequence direction was adjusted. Barcodes were matched exactly, and two nucleotides allowed to be mismatched in primer matching. The raw reads were deposited into the NCBI Sequence Read Archive database (accession number: SRP427453).

Then, the optimized sequences were clustered into operational taxonomic units (OTUs) using UPARSE 7.1 with a 97% sequence similarity level. The most abundant sequence for each OTU was selected as the representative sequence. OTUs with fewer than 100 total reads across all samples were excluded from further analysis to reduce noise and improve data reliability.

The taxonomy of each OTU representative sequence was analyzed by RDP Classifier version 2.2 [21] against the 16S rRNA database (e.g. Silva v138) using a confidence threshold of 0.7. The OTU table was manually filtered, i.e. removing OTUs that aligned to chloroplast and mitochondria sequences. To minimize the effects of sequencing depth on α- and β-diversity measure, the number of 16S rRNA gene sequences from each sample were rarefied to the minimum sample size, which still yielded an average Good’s coverage of 99.95%. Then, the sequences for each OTU were compared to those in GenBank using the BLASTn. Annotation and sequence alignment results for representative OTUs, and the abundance of OTUs across samples/replicates are shown in Table S2.

The Majorbio Cloud platform (https://cloud.majorbio.com) was used for bioinformatic analysis of the *D. gallinae* microbiota. Species composition, α-diversity, and β-diversity were analyzed for the E/AF/AM and EA/SA groups. Venn diagram analysis was performed for the E/AF/AM group, and the linear discriminant analysis (LDA) effect size (LEfSe) (http://huttenhower.sph.harvard.edu/LEfSe) was performed to identify the significantly abundant taxa (OTU) of bacteria among the EA/SA (LDA score > 3.5, *P* < .05). The α-diversity indices such as observed OTUs, Shannon index, and Abundance-based Coverage Estimator (ACE) index were calculated using Mothur v1.30.1. The similarity among the microbial communities in different samples was determined by principal co-ordinates analysis (PCoA) based on Bray–Curtis dissimilarity using the Vegan v2.5-3 package. Venn diagram analysis was conducted in R statistical software (version 3.0.3) using the vegan package.

### PacBio sequencing of full-length 16S rRNA gene

To achieve more accurate classification of unclassified Rhizobiaceae symbionts (abbreviated as un-Rhi), sample C1 (described in detail in the section on antibiotic treatment) containing a high abundance of un-Rhi was subjected to full-length sequencing of the 16S rRNA gene using the PacBio Sequel II sequencing platform. The extraction and purification of microbial community genome DNA from mites followed the same procedure as V3–V4 amplicon sequencing. Subsequently, the bacterial 16S rRNA genes were amplified using universal bacterial primers 27F and 1492R (Supplementary Table S1). Primers were tailed with PacBio barcode sequences to distinguish each sample. The amplification reaction system and conditions were the same as those used for V3–V4 amplicon sequencing. After electrophoresis, the PCR products were purified using AMPure® PB beads (Pacific Biosciences, CA, USA) and quantified using Qubit 4.0 (Thermo Fisher Scientific, USA).

Purified products were pooled in equimolar and DNA library was constructed using the SMRTbell prep kit 3.0 (Pacific Biosciences) according to PacBio’s instructions. Purified SMRTbell libraries were sequenced on the PacBio Sequel IIe System (Pacific Biosciences) by Majorbio Bio-Pharm Technology Co. Ltd. (Shanghai, China). High-fidelity (HiFi) reads were obtained from the subreads, which were generated using circular consensus sequencing via SMRT Link v11.0.

HiFi reads were barcode identified and length filtered, and the sequences with a length <1000 or >1800 bp were removed. The optimized-HiFi reads were clustered into OTUs using UPARSE 7.1 with 97% sequence similarity level. The most abundant sequence for each OTU was selected as the representative sequence. The OTU table was manually filtered, i.e. chloroplast sequences in all samples were removed. The taxonomy of each OTU representative sequence was analyzed by RDP Classifier version 2.2 against the 16S rRNA gene database (e.g. Silva v138) using a confidence threshold of 0.7. Annotation and sequence alignment results for representative OTUs are shown in Table S3. The sequence dates of un-Rhi (*Bartonella* A) and *Bartonella* B have been uploaded to the NCBI database (accession numbers: OQ653472.1 and OQ683804.1).

### Phylogenetic analysis of unclassified Rhizobiaceae

Sequences of un-Rhi 16S rRNA genes from *D. gallinae* were compared to available sequences from GenBank, based on sequence similarity to the un-Rhi found in various arthropod species. The sequences were aligned using ClustalW and a maximum-likelihood phylogenetic tree was constructed using the Kimura 2-parameter (K2) model with gamma distributed with invariant sites (G + I) [16]. Reliability of the tree was tested using bootstrap analysis (1000 replicates) with bootstrap values indicated on the tree. All phylogenetic analyses were performed using MEGA version 7.

### Culturing and isolation of bacteria

For the isolation of bacteria in *D. gallinae*, samples of mites at different developmental stages were collected, including eggs (E), larvae (L), nymphs (N), fed adult females (AF), and adult males (AM). The samples were surface cleaned for mites or eggs following the described procedure above. Then, the mites (*n* = 20) or eggs (*n* = 200) were transferred into a 1.5-mL microcentrifuge tube containing 100 μL of sterile 1× phosphate-buffered saline (PBS) and homogenized with a sterile homogenizer. The homogenized lysate was adjusted to a suitable concentration where bacterial colonies were individually and uniformly distributed on the medium for isolation of bacteria. An aliquot of 100 μL dilution was plated on LB Agar, Nutrient Agar (NA), or Brain-Heart Infusion Agar (BHIA), respectively. The plates were then incubated at 37°C for 24 to 48 h under aerobic and anaerobic conditions, respectively. Bacterial colonies obtained on plates were differentiated morphologically based on colony shape, size, color, margin, opacity, elevation, etc. Morphologically distinct colonies were selected from primary plates and subcultured on the corresponding plates until a pure colony was obtained. All isolated bacterial colonies were stored individually in 25% glycerol at −80°C before further molecular identification.

For the isolation of intestinal bacteria, the freshly fed adult females collected from rearing system were washed with Milli-Q water before being placed on a double-sticky tape for midgut dissection, as described previously [15]. Briefly, immobilized mites were covered with a 50-μL drop of sterile PBS at pH 7.2. The mites were decapitated, and their guts were expelled from the body cavity using a gentle pressure from the other side of their bodies. Five gut organs were transferred into 200 μL of sterile PBS and then homogenized using vortex. Fifty microliters of each homogenate was plated on Tryptic Soy Agar, NA, and BHIA plate and incubated at 37°C for 24 h. Morphologically distinct colonies were selected and subcultured on the corresponding plates until a pure colony was obtained.

For molecular identification of isolated bacteria, the DNA was extracted from each pure culture using the Bacterial Genomic DNA Extraction Kit (Solaibio, Guangzhou, China) according to the manufacturer’s instructions. The 16S rRNA gene fragment, ~1500 bp in size, was amplified using primers 27F and 1492R in a reaction volume of 20 μL, containing 10 μL 2× PCR Mix (Tsingke Biotech, Beijing, China), 1 μL Primer F, 1 μL Primer R, 2 μL DNA, and 6 μL sterile ddH_2_O [22]. Gene fragment was amplified according to the following cycle: initial denaturation at 94°C for 5 min, followed by 30 cycles of denaturation at 94°C for 30 s, annealing at 55°C for 30 s, and extension at 72°C for 1 min. The final extension was at 72°C for 10 min. The amplified product was visualized on a 1% agarose gel containing ethidium bromide using a UV transilluminator. The PCR products were sent to Shenggong (Sangon Biotech, Shanghai, China) for 16S rRNA genes sequencing using the Sanger method.

### Hemolytic activity of isolated bacteria

Hemolytic activity of all isolates was verified by blood agar plate. Initially, fresh chicken blood was aseptically collected from healthy chickens and treated with a heparin concentration of 20 U/mL to prevent coagulation. The blood was then combined with sterilized LB agar medium to produce a 5% blood agar plate. Then, overnight cultures of 82 isolates from *D. gallinae* were streaked (*Bacillus* sp., *Pseudomonas* sp., *Enterococcus* sp., *Clostridium* sp.) or spotted (*Proteus* sp.) on blood agar plates, with *Clostridium* sp. being incubated anaerobically at 37°C and 50% RH, and the other strains being incubated aerobically at 37°C and 50% RH. After 24–48 h, the formation of a lysis zone (halo) around the bacteria was observed to determine the hemolytic activity of the isolates. Isolates with a 1–2-mm-wide green zone around the colony were recorded as α-hemolysis, while those with a clear 2–4-mm-wide zone were denoted as β-hemolysis, and those that did not produce any zone around the colony were referred to as no hemolysis [23].

### Hemolytic activity assay of mite homogenate

To determine the role of symbiotic bacteria in the hemolysis of *D. gallinae* after a blood meal, antibiotic treatment was employed. Each group contained 100 blood-fed adult female mites that were homogenized with a sterile homogenizer in 1.5 mL of PBS at pH 7.2. The resulting homogenate suspension was divided equally into two portions: one portion was treated with oxytetracycline (OTC) to achieve a final concentration of 0.5 mg/mL, and the other portion was treated with an equal amount of PBS as a control. Subsequently, both portions were incubated at 4°C for 3 h. At the end of the incubation period, hemolytic activity assays of the homogenate were performed according to a previously described method [24] with minor modifications. In brief, chicken blood was collected using heparinized syringes to prevent clotting. The heparinized blood was promptly centrifuged at 1500×*g*, and the supernatant was removed. Red blood cells (RBCs) were washed at least three times in isotonic PBS at pH 7.2. The RBCs were then suspended in an isotonic buffer (20 times the volume) to achieve a 5% RBC suspension. Subsequently, 125 μL of the RBC suspension was added to a 1.5-mL centrifuge tube, followed by 20 μL of OTC pre-incubated or unpre-incubated mite homogenate and 105 μL of PBS to a total volume of 250 μL. The tubes containing this mixture were placed in a shaking metal bath at 30°C. At 5 h and 10 h, the mixture was centrifuged at 1500×*g* for 3 min, and 100 μL of the supernatant was transferred to a 96-well plate. The absorbance of this solution was measured at 570 nm. After the first absorbance detection, the 100 μL supernatant was transferred back into the mixture, mixed, and incubation was continued for second determination. Each group consisted of three replicates.

### Antibiotic treatments

To explore the function of symbiotic bacteria in *D. gallinae*, three antibiotics (oxytetracycline, OTC; tetracycline hydrochloride, TC; rifampicin, RIF) were used to intervene the symbiotic bacteria in *D. gallinae*, respectively. The OTC injection is a commercial reagent with a concentration of 200 mg/mL (Ouke Animal Pharmaceutical Co., Ltd, Jiangsu, China). TC (purity: 99.5%) and RIF (purity: 98%) (Macklin Biochemical Technology Co., Ltd, Shanghai, China) were dissolved in 40% *N*-*N*-dimethylformamide to formulate a solution with a concentration of 200 mg/mL.

Eight-week-old chickens were randomly allocated to four groups: OTC group, TC group, RIF group, and control group (C). Each group consisted of three replicates (cages), with each cage housing three chickens. Chickens in the antibiotic treatment groups received antibiotics through multipoint intramuscular injections into the pectoral muscles, with a dosage of 200 mg/kg based on pre-experiment. Control chickens were administered a placebo solvent without the inclusion of any drug. After the treatment, the chicks were returned to their cages. Two hours later, 800 starved female mites were introduced to the chicks in each cage to allow the mites to feed. After a 6-h period of darkness, all engorged female mites (~400 mites per cage) were recovered from the trap tubes, cages, and storage box after the chicks were removed. The chicks’ general health was monitored daily to ensure safety and health during the study.

To observe changes in appearance within the digestive tracts of the mites, 50 engorged mites were selected for examination. For the evaluation of blood digestion rate, 100 mites were placed into a 100-mL centrifuge tube and weighed by a microbalance (Sartorius MSA125P-1CE-DI, Gottingen, Germany). Another 100 mites were utilized to assess the reproductive capacity, including oviposition rate, fecundity, and hatching rate of their produced eggs. These mites were individually transferred to wells in 96-well plates, which were then kept in an incubator at 30°C and 75% RH. The mite oviposition and egg hatching were examined daily under a stereomicroscope (SteREO-Discovery V12; Carl Zeiss, Jena, Germany) for 5 days. Additionally, 100 mites were used to observe embryonic development where the mites were placed in an incubator at 30°C and 75% RH for 24 h to allow them to lay eggs. Freshly laid eggs were collected into a flat dish and embryo development was examined daily under a stereomicroscope (SteREO-Discovery V12; Carl Zeiss, Jena, Germany) for 4 days.

The digestion rate, oviposition rate, fecundity, hatching rate, and proportion of egg laid were calculated by employing the following formulae:

Digestion rate = (Average weight after feeding − Average weight after digesting period) / Average weight gain after feeding × 100%.

Oviposition rate = No. of mites laying eggs / No. of mites × 100%.

Fecundity = No. of eggs / No. of mites laying eggs.

Hatching rate = No. of larvae / No. of eggs × 100%.

Proportion of egg laid = No. of eggs laid on a certain day / Total number of eggs laid × 100%.

The effects of antibiotics on the fitness of *D. gallinae* were evaluated by comparing administered mites and control ones.

In order to rule out the off-target effect of antibiotics, phenotypic recovery tests were conducted in antibiotic-treated mites. The mites initially fed on OTC-treated chicken to eliminate symbiotic bacteria during the first feeding were subsequently continuously fed three times with un-treated chickens, and mite reproductive performances, including oviposition rate, fecundity, and egg hatching rate, were observed after each feeding.

The effect of antibiotics on erythrocyte lysis was detected by counting RBCs in the intestine of mites. The enumeration of RBCs was done at 0, 6, 12, 24, 36, 48, and 60 h after blood feeding (ABF). Control and OTC-treated mites were transferred to slides, and a drop of saline was added. The shells of mites were gently punctured with an acupuncture needle to allow the intestinal contents to be discharged, and the intestinal contents were diluted in 100 μL saline. Subsequently, 10 μL suspension was transferred to a hemocytometer. The number of RBCs obtained in treatment and control group was compared to that obtained from females dissected immediately ABF [25]. Cell counting was performed on 15 mites in each group.

The effect of antibiotic treatment on the degradation of hemoglobin in mites was detected by SDS-PAGE. On the second and third days after blood feeding, the mites in the control and OTC-treated groups were transferred to a 1.5-mL tube, and 100 μL 0.1 M PBS (pH 7.8, containing protease inhibitors) was added and homogenized on ice. The homogenate was centrifuged at 12 000 rpm at 4°C for 15 min, and the protein in the supernatant was detected by SDS-PAGE.

### The effect of antibiotics on the bacterial composition of mites

To analyze the effect of antibiotics on the bacterial composition of mites, the optimal antibiotic (OTC) was used. The mites in the control group (C) and treatment group (T) were collected for microbial community analysis on the first day after engorgement, with each sample comprising 100 individuals. Amplicon sequencing was then performed following the described procedure above.

### Quantitative polymerase chain reaction assay

To quantify the number of 16S rRNA copies of bacteria in the samples, the universal primers Com1 and 769R (Supplementary Table S1) [26] were utilized. To quantify the number of *Kocuria* and *Bartonella* A 16S rRNA copies in the samples, the specific primers for *Kocuria* and *Bartonella* A (Supplementary Table S1) were employed.

Amplifications were carried out using an ABI 7500 Real-Time PCR system (Applied Biosystems, Foster City, CA, USA) utilizing the SYBR Green method. qRT-PCR was performed using TransStart® Tip Green qPCR SuperMix (+Dye I) (TransGen Biotech, Beijing, China) following the manufacturer’s protocol. The qPCR cycling conditions consisted of an initial step of 95°C for 3 min, followed by 40 cycles of 95°C for 15 s and 60°C for 1 min. Baseline and threshold calculations were conducted using the StepOnePlus software version 2.3 (Thermo Fisher Scientific). Standard curves were established using targets cloned into an *Escherichia coli* plasmid, with serial dilutions ranging from 10^8^ to 10^2^ gene copies per reaction. The assays exhibited coefficient of determinations (*R*^2^) for the log regression of Ct values versus DNA dilutions of 0.99, efficiencies exceeding 92%, and sensitivities ranging from 1 to 100 gene copies. The resulting data were standardized by recalculating per one mite.

### Prevalence of *Kocuria* and *Bartonella* A in *D. gallinae*

In order to detect the prevalence of two core bacteria (*Kocuria* and *Bartonella* A) in *D. gallinae*, 72 engorged adult female mites were randomly selected from the rearing system, and DNA was extracted from the whole body of each adult mite using a DNeasy Blood and Tissue Kit (CWBIO, Jiangsu, China) with 30 μL of EB buffer. PCR was conducted using Es Taq MasterMix (CWBIO). The specific primers were designed by Primer Premier 5 (Supplementary Table S1). The PCR amplification of the 16S rRNA gene of *Kocuria* and *Bartonella* A was performed as follows: initial denaturation at 95°C for 5 min, followed by 30 cycles of denaturing at 95°C for 30 s, annealing at 60°C for 30 s and extension at 72°C for 1 min, and a single extension at 72°C for 10 min, and ending at 12°C. The amplicon was verified using 1% (w/v) agarose/Tris acetate-EDTA (TAE) gel.

### Isolation and morphological characterization of *Bartonella* A

To isolate *Bartonella* A from *D. gallinae*, the initial step was to sterilize the surface of fed adult females with 75% ethanol for 3 h. Subsequently, the mites were washed three times with sterile water. Then, the mites (*n* = 15) were homogenized in 1.5-mL tubes containing 300 μL of sterile PBS. After appropriate dilution, the homogenate was plated onto chocolate agar supplemented with defibrinated sheep blood (10%, vol/vol). The plates were incubated in a carbon dioxide incubator (BPN-50CH; Shanghai Yiheng Scientific Instrument Co., Ltd) at 35°C under 5% CO_2_ for 5–7 days. Bacterial colonies suspected of *Bartonella* based on colony morphology were subjected to PCR amplification of the 16S rRNA gene, and the PCR products were then verified by Sanger sequencing. Colonies belonging to *Bartonella* A were then subcultured onto the same medium to achieve purity.

Cell morphology of *Bartonella* A was examined using transmission electron microscopy (TEM). For negative staining [27], the bacterial suspension was dipped into a copper grid coated with Formvar–carbon film, stained with 1% uranyl acetate for 3 min, and then observed with FESEM (SU-8010, Japan).

### Whole-genome sequencing and comparative genomic analysis of *Bartonella* A

The genomic DNA of *Bartonella* A was extracted using the bacterial genomic DNA purification kit (Tiangen Biochemical Technology, Beijing, China). After the assessment of DNA purity and integrity through agarose gel electrophoresis, genome sequencing was conducted using PacBio and Illumina PE150 platforms. The data were then employed for genome assembly through Canu (https://github.com/marbl/canu/) [28]. By utilizing the web service (http://www.bioinformatics.org/sms/), the G + C content of the genomic DNA was calculated.

For species-level taxonomic identification of *Bartonella* A at the genome level, a comparison was made with 32 complete *Bartonella* genomes (Supplementary Table S4) for further analysis. A phylogenetic tree based on the whole genome was reconstructed using the TYGS platform (https://tygs.dsmz.de/) [29]. The average nucleotide identity (ANI) was computed by analyzing genomes from different *Bartonella* species using the IPGA online tool (https://nmdc.cn/ipga/) [30]. Moreover, we also conducted *in silico* DNA–DNA hybridization (DDH) between *Bartonella* strains using GGDC (https://ggdc.dsmz.de/) software [31].

### Genome annotation and functional pathway analysis

Coding-gene prediction of the assembled *Bartonella* A was performed using GeneMarkS [32] program (http://topaz.gatech.edu/GeneMark/). Annotation of non-coding rRNAs was carried out using rRNAmmer [33], while tRNAs were identified using tRNAscan-SE [34]. Additionally, snRNAs were predicted by BLAST against the Rfam [35] database. Functional annotation of the protein-coding genes was conducted by searching against the KEGG database [36], SwissProt (http://uniprot.org), and COG [37] (E-value <1e−5, minimal alignment length percentage >40%). Based on the genome annotation results, we inspected the metabolic pathways for essential amino acids, B vitamins, and cofactors and constructed the corresponding KEGG pathway map. The absence of genes in pathways was confirmed through tblastn searches conducted on the *Bartonella* A genome.

### Statistical analysis

All statistical analyses were conducted using GraphPad Prism 8.0.1 Software. All data were analyzed with a Shapiro–Wilk normality test followed by parametric or nonparametric analysis depending on whether data were normally distributed. For parametric analysis, Student’s *t* test was used for the comparison of two groups, while one-way analysis of variance (ANOVA) with Tukey’s test was used for multiple treatment comparisons. For nonparametric analysis, we ran Mann–Whitney test for comparisons of two treatments or Kruskal–Wallis test for multiple treatment comparisons. Statistically significant differences were indicated with asterisks as follows: ^*^P < .05, ^*^^*^P < .01, ^*^^*^^*^P < .001, and no labeling represented no difference between groups.

## Results

### *Dermanyssus gallinae* mites host considerable midgut bacterial microbiota across stages, dominated by *Kocuria* and *Bartonella* A species in blood-feeding females

**Figure 1:**
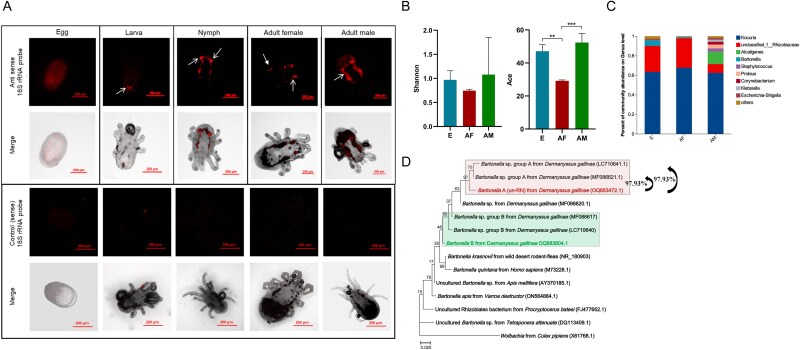
Location and composition analysis *D. gallinae* bacterial microbiota across stages. (**A**) Localization of bacterial microbiota in *D. gallinae*. Fluorescence *in situ* hybridization analysis of the bacteria in *D. gallinae* at different developmental stages using the universal anti-sense 16S rRNA probe, and a sense probe served as a negative control. The merged image showing overlap of bacteria channel on bright field. The area indicated by the white arrow is the ceca of the mites. Different exposure times were used to obtain observable fluorescence intensity. (**B**) Shannon and ACE indices for bacterial diversity in different groups. Asterisks indicate the statistical significance: ^*^P-value <.05; ^*^^*^P-value <.01; ^*^^*^^*^P-value <.001 in the one-way ANOVA. (**C**) Whole profiles of the relative abundances of bacterial genus in *D. gallinae* across stages; all OTUs that accounted for <1% were combined “other.” EA and SA indicate the engorged adult female and starved adult female, respectively. (**D**) Phylogenetic analysis of the full-length 16S rRNA sequences of *Bartonella* A and related species. A maximum-likelihood phylogeny (model K2 + G + I) inferred from 1446 nucleotide sites are shown with bootstrap probability at each node. The color-labeled sequences are those obtained in this study. OTU of *Bartonella* from *D. gallinae* are shaded with light green and light red. Numbers in brackets indicate NCBI accession numbers.

To reveal the bacterial distribution over mite’s interior, we visualized the microbiota with fluorescently labeled universal bacterial 16S rRNA probe (EUB338-Cy5). The results showed that bacteria were distributed evenly throughout the eggs, while in the nymph, adult female, and male mites, a high prevalence of bacteria was detected in the ceca (Fig. 1A), a morphological structure within the digestive organ that plays a crucial role in nutrient digestion and absorption. Due to the agenesis of the larvae intestine, only minimal fluorescence was observed in this developmental stage. This localization pattern suggests that the bacterial community in *D. gallinae* may play an important role in digestion and metabolism of nutrients. To gain a comprehensive understanding of bacterial diversity in blood-feeding mites (adult females, AF) and non-feeding stages (eggs, E; adult males, AM), we subjected these samples to 16S rRNA gene amplicon sequencing. The 16S rRNA amplicon sequencing generated a median of 58 307 reads per sample, with a range from 48 045 to 71 847 reads. The corresponding base counts ranged from 19 624 292 to 30 347 532 bases (Supplementary Table S5). Two indices, the Shannon index for bacterial diversity and the ACE index for species richness, were employed to evaluate the bacterial diversity in three groups. As depicted in Fig. 1B, the egg and adult male groups exhibited higher diversity compared to the adult female group, although there was no statistical difference. Regarding species richness (Fig. 1B), the adult male group displayed the highest index, followed by the egg group, while the adult female group had the lowest ACE index. Although there was variation in the bacterial community composition among individual samples, the bacterial community composition of adult females partially resembled that of eggs (Supplementary Fig. S2). Venn diagram analysis indicated 39 bacterial OTUs were shared by the eggs, adult females, and adult males of *D. gallinae* (Supplementary Fig. S3). Micrococcaceae was the dominant family across all groups, with an average relative abundance of 64.52 ± 2.32%. Rhizobiaceae followed with an average relative abundance of 24.08 ± 10.63% (Fig. 1C). Among these three groups, the most abundant genus was *Kocuria* sp. with an average relative abundance of 64.52 ± 2.32%. Un-Rhi had an average relative abundance of 21.89 ± 9.24%. To specify the un-Rhi sequence, the full-length 16S rRNA gene sequences were obtained and sequenced, which then clustered with *Bartonella* group A previously identified in *D. gallinae*, and showing over 97% similarity (Fig. 1D). For simplicity of presentation, this bacterium was further referred to as *Bartonella* A. Additionally, another type of *Bartonella* was identified in the mites, closely related to *Bartonella* group B, which had previously been detected in *D. gallinae* mites [4, 16]. This bacterium is further referred to as *Bartonella* B. Multiple sample screening further indicated that the infection rate of *Kocuria* in the mite population was 66.67% (48/72), while the infection rate of *Bartonella* A was 100% (72/72) (Supplementary Fig. S4). This result suggests that *Bartonella* A may be an obligate symbiont of *D. gallinae*.

### Blood-feeding reduces α-diversity with dominance of *Kocuria* and *Bartonella* A

**Figure 2:**
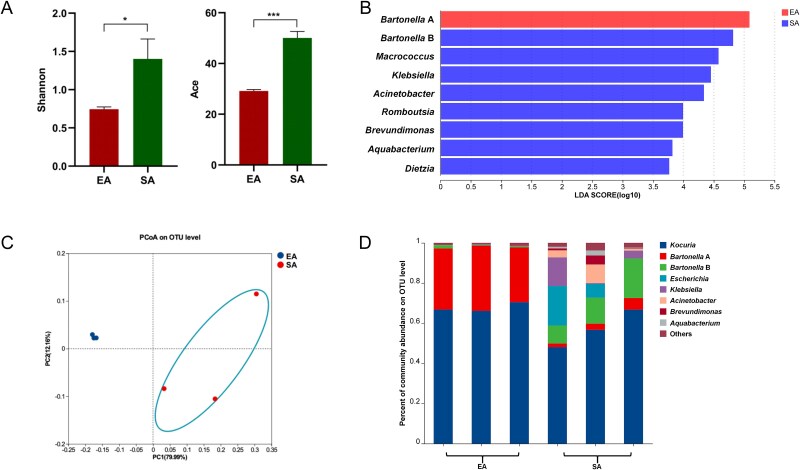
The effect of bloodmeal on the bacterial communities in *D. gallinae*. (**A**) Shannon and ACE indexes of *D. gallinae* bacterial microbiota were related to blood-feeding. Student’s *t* test. ^*^P < .05; ^*^^*^P < .01, ^*^^*^^*^P < .01. (**B**) The LDA effect size (LEfSe) algorithm with LDA scores ≥3.5 was applied to OTU tables to identify taxa that most effectively characterize each biological class. (**C**) The PCoA for bacterial community composition based on Bray–Curtis. (**D**) Whole profiles of the relative abundances of bacterial taxa in engorged and starved mites; all OTUs that accounted for <1% were combined “other.” EA and SA indicate the engorged adult female and starved adult female, respectively.

To assess bacterial diversity dynamics in response to blood-feeding, starved adult (SA) mites and freshly blood-engorged adult (EA) mites were subjected to 16S rRNA amplicon sequencing. The starved group was found to have a higher α-diversity compared to the engorged group (Fig. 2A). The linear discriminant analysis (LDA) identifies *Bartonella* A bacteria as the most abundant species in the freshly engorged mites (Fig. 2B). PCoA further demonstrates a significant separation between the bacterial communities of starved and engorged mites, indicating that blood feeding has a pronounced impact on the bacterial community composition of *D. gallinae* (Fig. 2C). The main bacterial taxa in starved mites include *Kocuria*, *Bartonella* A, *Bartonella* B, *Escherichia*, *Klebsiella*, *Acinetobacter*, and *Brevundimonas*. In engorged mites, in contrast, *Bartonella* B, *Escherichia*, *Klebsiella*, *Acinetobacter*, and *Brevundimonas* were no longer dominated, while *Bartonella* A constituted a notable fraction of the microbiota (Fig. 2D). This shift in bacterial composition suggests that *Bartonella* A bacteria may play a significant role in the mite’s physiology and vital activities following a blood meal.

### Culturable bacteria from *D. gallinae* exhibit hemolytic activity *in vitro*

**Figure 3:**
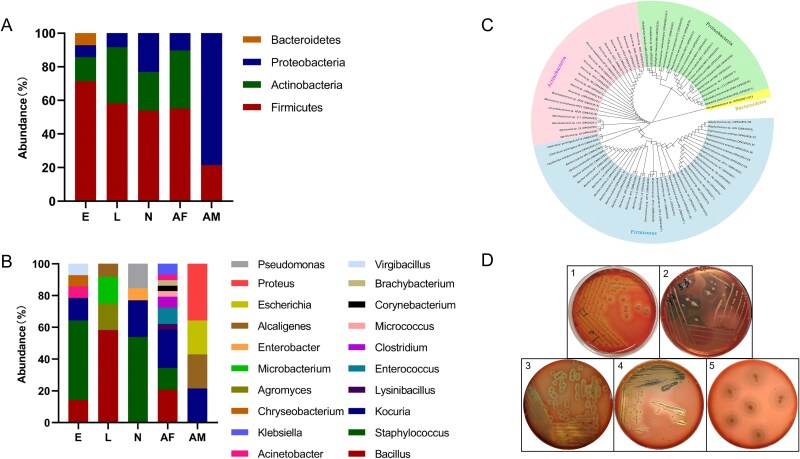
Analysis of culturable bacteria of *D. gallinae*. (**A**) Relative abundance of phylum. (**B**) Relative abundances of genera. (**C**) Phylogenetic analysis of 16S rRNA sequences (~1500 bp) of all isolated bacteria using neighbor-joining (NJ) method with 1000 bootstrap replicas. The first part of the sequence ID denotes the bacterial species. The second part of the sequence ID indicates from which life stage the bacteria were isolated. The third part of the sequence ID is the GenBank accession number. Only the significant bootstrap values (>50%) from 1000 replicates are shown on the nodes. In all three figures, E, L, N, AF, and AM indicate the egg, larva, nymph, adult female, and adult male, respectively. (**D**) Hemolytic activities of bacteria isolated from *D. gallinae*. 1, *Bacillus* sp.; 2, *Clostridium* sp.; 3, *Pseudomonas* sp.; 4, *Enterococcus* sp.; 5, *Proteus* sp. halos correspond to the regions of erythrocytes lysis.

To complement the 16S rRNA amplicon sequencing data, bacteria were isolated and cultured from *D. gallinae* mites, and identified based on culture-dependent 16S rRNA sequencing. A total of 82 bacterial strains were identified. The majority of the isolates (48.78%; 40/82) were categorized under the phylum Firmicutes. The other 42 isolates were from the Actinobacteria (26.83%; 22/82), Proteobacteria (23.17%; 19/82), and Bacteroidetes (1.22%; 1/82) phyla (Fig. 3A). The isolated and identified bacteria included 20 genera (Fig. 3B). The adult female had a higher diversity with 17 species belonging to 11 genera: *Bacillus*, *Lysinibacillus*, *Staphylococcus*, *Enterococcus*, *Clostridium* (including pathogenic bacterium *Clostridium perfringens*), *Kocuria*, *Micrococcus*, *Corynebacterium*, *Brachybacterium*, *Acinetobacter*, and *Klebsiella*. In contrast, fewer bacterial genera were found in other stages. The three genera *Bacillus*, *Staphylococcus*, and *Kocuria* were the most frequently isolated in all stages with the exception for adult males. The majority of bacterial strains (78.58%) isolated from eggs belonged to the genera *Bacillus*, *Staphylococcus*, and *Kocuria*, along with a small amount of strains from the genera *Lysinibacillus*, *Acinetobacter*, and *Chryseobacterium*. Among the larvae, over half of the isolates (58.33%) belonged to the genus *Bacillus*, with a few strains belonging to the genera *Agromyces*, *Microbacterium*, and *Enterobacter*. For nymphs, 76.93% of the isolates were classified into the genera *Bacillus* and *Kocuria*, while one belonged to the genus *Enterobacter*, and an opportunistic pathogen, *Pseudomonas aeruginosa*, was isolated. Several strains belonging to the genera *Proteus* and *Alcaligenes* were isolated from adult males, and other intestinal bacteria such as *Kocuria* sp., *Enterococcus* sp., and *Escherichia* sp. were also found (as shown in Fig. 3B). Additionally, culturing of midgut homogenate of adult *D. gallinae* female mites revealed the presence of *Staphyloccocus*, *Kocuria*, and *Escherichia* bacteria (Supplementary Fig. S5). Phylogenetic analysis confirmed a distant relationship between the bacteria isolated from mites at different developmental stages (Fig. 3C). Interestingly, approximately one-quarter of the isolates (23.17%, 19/82) showed strong hemolytic activity on blood agar plates and they belonged to the genera *Bacillus*, *Clostridium*, *Pseudomonas*, *Enterococcus*, and *Proteus*. The hemolytic activity of representative strains in each genus is shown in Fig. 3D and the corresponding hemolytic types are shown in Table S6.

### Dysbiosed *D. gallinae* mites lack the post-blood-feeding bloom of *Bartonella* A symbiont

**Figure 4:**
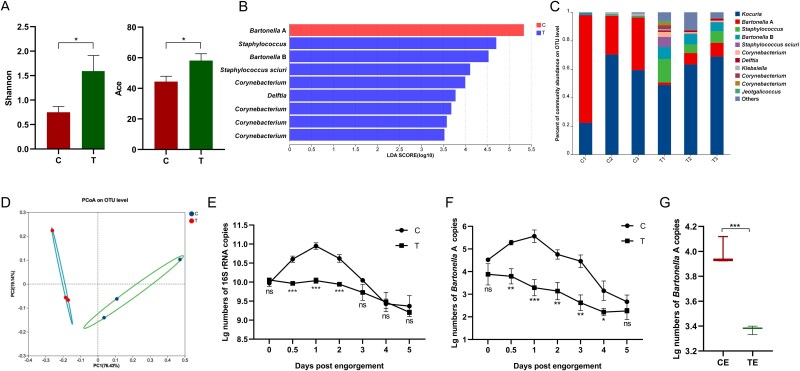
The effect of antibiotics treatment on the bacterial communities and abundance in *D. gallinae*. (**A**) Shannon and ACE indexes of *D. gallinae* bacterial microbiota. Student’s *t* test. ^*^P < .05. (**B**) The LDA effect size (LEfSe) algorithm with LDA scores ≥3.5 was applied to OTU tables to identify taxa that most effectively characterize each biological class. (**C**) Whole profiles of the relative abundances of bacterial taxa in control and treatment groups; all OTUs that accounted for <1% were combined “other.” (**D**) The PCoA for bacterial community composition based on Bray–Curtis. (**E**) The quantification of numbers of copies of total bacterial 16S rRNA gene in control and treatment groups. (**F**) Numbers of bacterial 16S rRNA gene copies obtained from adult females by *Bartonella* A specific primers. (**G**) Numbers of bacterial 16S rRNA gene copies obtained from eggs by *Bartonella* A primers. Asterisks indicate statistical significance: ^*^P-value <.05; ^*^^*^P-value <.01; ^*^^*^^*^P-value <.001 in the Student’s *t* test. C, control; T, OTC treatment; CE, control eggs; TE, treatment eggs.

To further elaborate on the potential roles of symbiotic bacteria in aiding erythrocyte lysis in the digestive tract of mites, dysbiosed mites were produced. Using OTC as the antibiotic to interfere with the microbial community composition of *D. gallinae*, the α-diversity of the treated group was significantly higher than that of the control group, indicating a disruption of the relatively concentrated microbial population of mites after blood feeding (Fig. 4A). The LDA identified a significant loss of the *Bartonella* A species in the treated group (Fig. 4B), which was co-dominating the control group (Fig. 4C). The PCoA confirmed a significant separation between the two groups, indicating a distinct shift in the microbial community composition due to antibiotic treatment (Fig. 4D), ultimately evidencing the success in production the dysbiotic mites.

The qPCR results further corroborated the impact of OTC treatment on the bacterial load in *D. gallinae*, clearly reporting the initial rise of the total bacterial load upon blood-feeding, with subsequent gradual decline after day 1 after blood-feeding in the control groups, with flat and decreasing bacterial load upon blood-feeding in dysbiosed mites (Fig. 4E). Using *Bartonella* A specific primers, an almost identical pattern was observed, confirming a severe depletion of *Bartonella* A symbiont in the dysbiosed mites (Fig. 4F). Conversely, *Kocuria* displayed insensitivity to OTC, and its bacterial load trend appeared to be opposite to that of *Bartonella* A (Supplementary Fig. S6). Additionally, the abundance of *Bartonella* A in the eggs laid by the OTC-treated mites was also significantly suppressed compared to the control group (Fig. 4G). Overall, the results indicated that antibiotic treatment disrupted the microbial community composition and reduced the abundance of symbiotic bacteria, particularly *Bartonella* A bacteria in *D. gallinae*.

**Figure 5:**
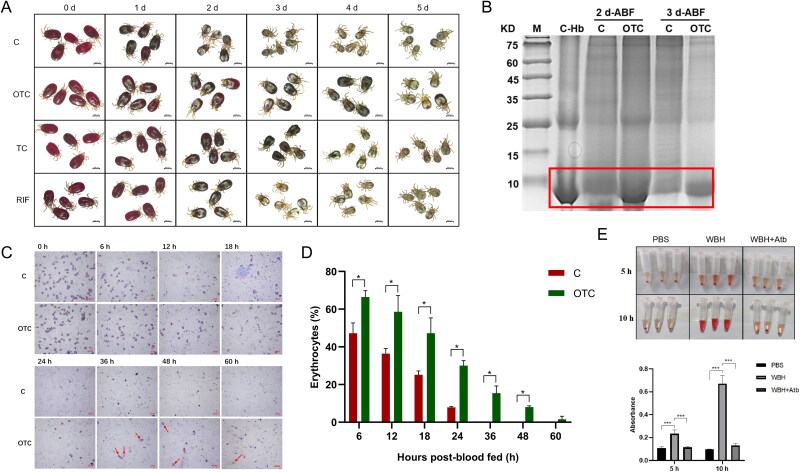
Effect of antibiotics treatment on blood digestion of *D. gallinae*. (**A**) Mite appearance at digestion periods. Engorged mites in control or treatment groups were collected and observed by stereomicroscope. Several mites were randomly selected from the surviving mites and observed on day 1 (1 d), day 2 (2 d), day 3 (3 d), day 4 (4 d), and day 5 (5 d) after collection. (**B**) SDS-PAGE of hemoglobin accumulation in mites after antibiotic treatment. M, standard marker; C-Hb, chicken hemoglobin. (**C**) Erythrocyte lysis in the intestines of mites observed under the microscope. (**D**) Statistical results of erythrocyte count, with the percentage of erythrocytes in the midgut expressed in relation to the total ingested by recently fed mite (0 h). C, control; OTC, oxytetracycline; TC, tetracycline; RIF, rifampicin; ABF: after blood feeding. (**E**) *In vitro* hemolytic test for mite homogenates. Asterisks indicate statistical significance: ^*^^*^^*^P-value <.001 in the Student’s *t* test. WBH, whole-body homogenate; WBH + Atb, whole-body homogenate pre-incubated with antibiotics.

### Dysbiosed mites display impeded hemolytic capacity upon blood-feeding

To investigate the impact of bacterial dysbiosis on the lysis and digestion of host red blood cells, we closely observed the mites following blood feeding. Control mites exhibited a gradual and rapid loss of their bright red coloration, indicative of the lysis and digestion of host red blood cells, while contemporaneous dysbiosed mites retained their vivid red hue, suggesting the containment of unlysed red blood cells within their bodies (Fig. 5A). This observation was further corroborated by reducing SDS-PAGE of whole-body mite homogenate, which clearly showed the retention of hemoglobin in dysbiosed mites (Fig. 5B). To investigate the bacterial contributions to blood hemolysis *in vivo*, we monitored the lysis of RBCs in mites during digestion following OTC treatment. Significant differences were observed in antibiotic-treated mites compared to controls at 0–24 h ABF. Indeed, from 36 to 60 h, a small number of intact RBCs were still present in the gut of antibiotic-treated females, whereas nearly no RBCs were visible in control group (Fig. 5C, D). Additionally, whole-body homogenate of mites induced erythrocyte lysis in the liquid suspension, and this activity could unequivocally be inhibited by the use of antibiotics (Fig. 5E). These results indicated that *D. gallinae* symbiotic bacteria were involved in hemolytic activity aiding blood digestion and that dysbiosed mites displayed compromised capacity to digest fully and timely host erythrocytes.

The differences in digestion rates between treatment groups and control groups were consistent with the visual appearance of the mites. As summarized in Table 1, the digestion rates of mites in the treatment groups were significantly lower than those in the control groups on days 1–3 post-feeding. For instance, by day 3, the digestion rate in the control group reached 90.8%, whereas the rates in the OTC-, TC-, and RIF-treated groups were 53.93%, 67.00%, and 84.07%, respectively. There was no noticeable difference in mite’s appearance at the initial engorgement stage when they were collected from antibiotic treatment groups or control groups. This suggested that the antibiotic treatment did not significantly affect the blood ingestion process of *D. gallinae*.

**Table 1:** Effects of antibiotic treatment on blood digestion of adult mites.

**Days after mite collection**	**Digestion rate (%)**			
	**C**	**OTC**	**TC**	**RIF**
1	53.03 ± 2.85^a^	16.6 ± 3.67^b^	23.97 ± 4.85^b^	18.8 ± 5.12^b^
2	86.43 ± 4.27^a^	41.07 ± 3.43^b^	53.90 ± 11.28^b^	49.13 ± 8.16^b^
3	90.8 ± 1.81^a^	53.93 ± 3.68^b^	67.00 ± 4.61^c^	84.07 ± 3.01^d^
4	94.33 ± 2.33^a^	67.33 ± 8.07^b^	81.53 ± 2.71^b^	91.87 ± 4.45^a^
5	99.57 ± 0.31^a^	81.87 ± 2.29^b^	97.83 ± 1.67^a^	94.57 ± 2.93^a^

The data are expressed as mean ± SD

^a, b, c, d^Values within the same row followed by different letters are significantly different

*Abbreviations*: C, control; OTC, oxytetracycline; TC, tetracycline; RIF, rifampicin

### Failure to digest host erythrocytes has profound negative consequences on the reproductive capacity of *D. gallinae* mites

**Figure 6:**
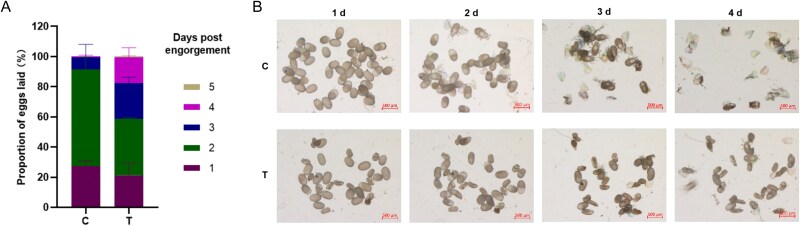
Effect of OTC treatment on reproduction of *D. gallinae*. (**A**) Daily proportion of eggs laid of adult female. Engorged mites in control or treatment groups were collected, the number of eggs laid per day was counted, and the daily egg production rate was calculated. The data are expressed as the mean ± SD. (**B**) Embryo development of mite. Newly produced eggs were randomly selected on day 1 (1 d) after mite engorgement, and embryo development was observed with a stereomicroscope on day 1 (1 d), day 2 (2 d), day 3 (3 d), and day 4 (4 d) after collection. C, control; T, OTC treatment.

To investigate the repercussions of impaired blood digestion, we monitored the fecundity of treated females and the viability of their laid eggs. The results, presented in Table 2, demonstrated significant differences in reproductive capacity between the antibiotic-treated group and the control group. In the OTC-treated group, all reproduction parameters were noticeably lower than those in the control group, and the TC-treated group had a lower egg hatching rate compared to the control group. Interestingly, OTC showed the strongest effects, and OTC treatment also caused a delay in oviposition (Fig. 6A). In the control group, mite oviposition was primarily concentrated within 1–2 days ABF. However, in the OTC-treated group, fresh eggs were still being produced up to 4 days after the blood meal. To assess embryonic development, the appearance and hatching of eggs were evaluated. The majority of eggs laid by mites in the control group successfully hatched on day 4 after collection (Fig. 6B). In contrast, some eggs laid by antibiotic-treated mites exhibited atypical appearances, such as being shriveled, and ultimately failed to hatch (Fig. 6B). Collectively, these results confirmed that antibiotic treatment significantly reduced the reproductive capacity of *D. gallinae*, negatively affecting oviposition, egg hatching, as well as embryonic development. The presence of symbiotic bacteria appears to play a crucial role in the blood digestion, which directly hinders the swift reproductive success of the mites, i.e. the most troublesome feature of *D. gallinae*’s impact on poultry farms.

**Table 2:** Oviposition rate (%), fecundity, and egg hatching rates (%) of adult female mites.

**Reproduction parameters**	**C**	**OTC**	**TC**	**RIF**
Oviposition rate (%)	97.29 ± 1.24^a^	64.93 ± 20.08^b^	94.65 ± 4.05^ac^	94.68 ± 4.01^ac^
Fecundity	4.06 ± 0.62^a^	1.05 ± 0.4^b^	3.75 ± 0.2^ac^	3.17 ± 0.54^cd^
Hatching rate (%)	98.58 ± 1.34^a^	22.54 ± 13.19^b^	73.76 ± 13.76^c^	85.3 ± 10.98^ac^

The data are expressed as the mean ± SD

^a, b, c, d^Values within the same row followed by different letters are significantly different

*Abbreviations*: C, control; OTC, oxytetracycline; TC, tetracycline; RIF, rifampicin

In order to rule out the off-target effect of antibiotics, phenotype recovery tests of mites were conducted. As shown in Table S7 and Fig. S7, the second feeding did not immediately restore mite’s reproductive performance to normal levels although a marginal enhancement in reproductive performance was observed, and total bacteria (16S) proliferate well from day 3 post-engorgement. The core bacteria, *Bartonella* A, are then responsive to blood meals in subsequent feedings (third and fourth), and proliferated immediately after feeding (0.5 days). These results indicated that *Bartonella* A seemed to be not fully eliminated by the OTC treatment; only its abundance was significantly reduced and seemed to recover from third feeding on and was positive responding to blood feeding by proliferation. Consistently, the mite’s reproductive performance was fully restored to normal levels by the fourth feeding. This result suggested that the antibiotic’s primary target was bacteria, and the temporal requirement was needed for re-constructing the colony following the withdrawal of the antibiotic.

### *Bartonella* A, isolated from *D. gallinae*, exhibited a reduced genome and a lower GC content, and was identified as a novel species

**Figure 7:**
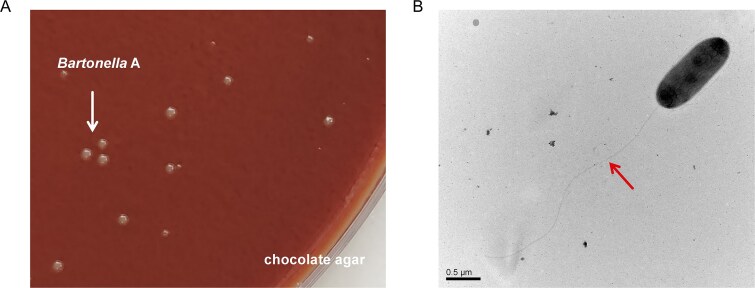
Morphological observation of *Bartonella* A. (**A**) *Bartonella* A colonies (arrow) grown on chocolate agar. (**B**) Transmission electron micrographs of negatively stained cells of *Bartonella* A isolates. Flagella-like structures were indicated with arrows. Scale bars indicate 0.5 μm.

Considering the significant roles of *Bartonella* A, we tried to isolate and characterize the bacterium. It was found that *Bartonella* A could be cultivated on chocolate agar (Fig. 7A). TEM inspection revealed that *Bartonella* A cells were rod shaped and had a two-layered cell envelope, which is a typical trait of Gram-negative bacteria (Fig. 7B). Cells were 0.5 μm in width and 1.3 μm in length, and possessed flagella-like structures.

Using PacBio sequencing, we *de novo* assembled the genome of *Bartonella* A, finding the genome was assembled into a 1 398 791-bp circular chromosome, which was predicted to encode 1411 protein-coding genes, 38 tRNAs, 6 rRNA, and 3 sRNA (Supplementary Fig. S8, Table S8). The GC content of the genome was 31.13%. The genome sequence of *Bartonella* A has been deposited in the NCBI (GenBank accession: CP166769). These results show that the genome of *Bartonella* A has undergone significant reductions and exhibits a lower GC content compared to other *Bartonella* species or strains (Supplementary Table S9), further indicating that *Bartonella* A is an obligate symbiont in *D. gallinae* [36, 37].

By comparing the *Bartonella* A isolate with 32 complete *Bartonella* genomes retrieved from the NCBI Genome database, phylogenomic relationships based on the whole nucleotide sequences were reconstructed (Supplementary Fig. S9). *Bartonella* A was labeled as an individual species cluster. The whole-genome ANI of *Bartonella* A was calculated in relation to phylogenetically related species, and the values between strains ranged from 64.93% to 66.87% (Supplementary Table S10). All these values were below the respective thresholds of 95% for ANI to delineate distinct species [38]. Additionally, DDH similarities have also been used to determine the species affiliation of strains, and it has been generally accepted that a 70% DDH value is proposed as a species boundary [39]. The results showed that the values between *Bartonella* A and other closely related species ranged from 22.80% to 43.40% for DDH (Supplementary Table S10), which was below the recommended threshold of 70% for prokaryotic species delineation. These data provide strong evidence that *Bartonella* A is a new species within the genus *Bartonella*. We suggest to name this newly discovered species as *Bartonella kongi* sp. nov., in honor of Fanyao Kong, a Chinese parasitologist.

### Identification of B vitamins and essential amino acid synthetic pathways from *Bartonella* A genome

**Figure 8:**
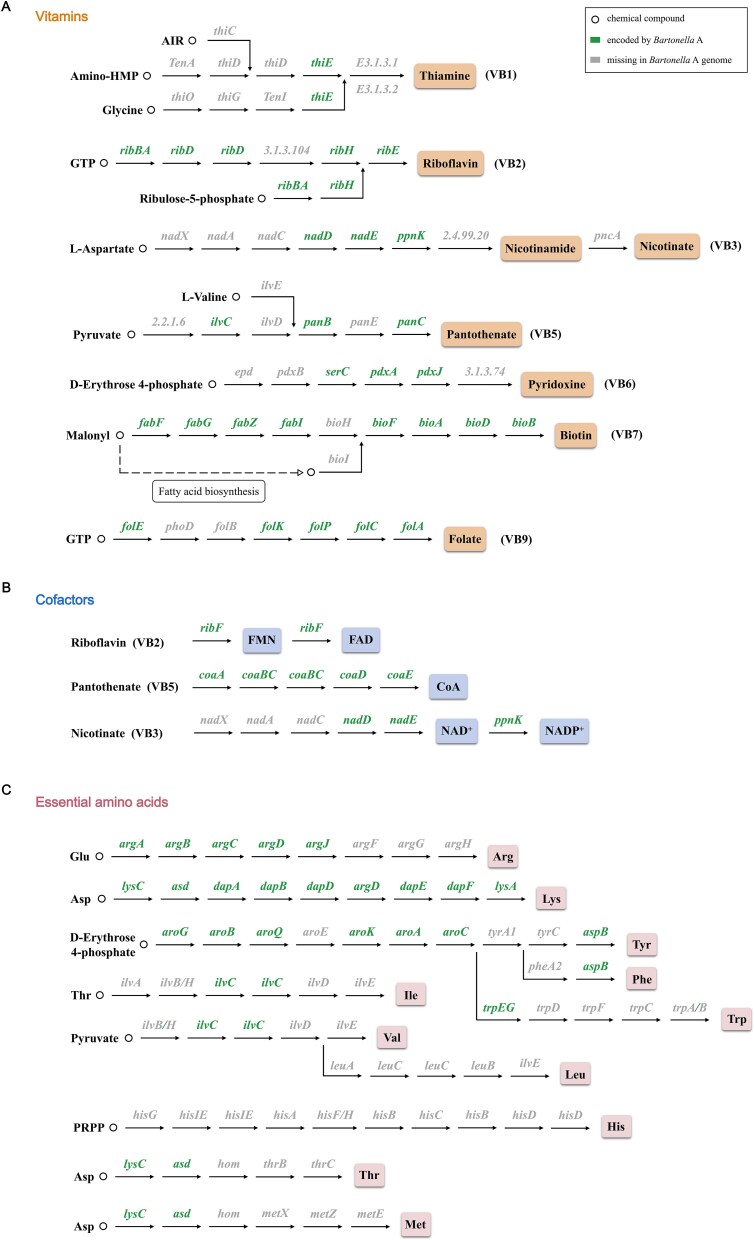
Biosynthetic pathways for synthesis of (**A**) vitamins, (**B**) cofactors, and (**C**) essential amino acids in *Bartonella* A genome. Circles represented compounds in synthetic pathways.

Previous research has indicated the importance of symbionts in supplying essential amino acids and B vitamins, which are necessary for the development and survival of insect hosts [40, 41]. The significance of vitamin B and cofactors supplementation by symbionts has been well recognized, especially in blood-feeding arthropods [42]. Therefore, in order to ascertain if *Bartonella* A possesses the similar ability, we reconstructed its vitamin and cofactor biosynthesis pathways. As shown in Fig. 8A and B, the *Bartonella* A genome has conserved genes involved in the biosynthesis of B vitamins and cofactors. For instance, it maintains the nearly complete biosynthesis pathway for riboflavin (5/6 genes present) and biotin (8/9 genes present), as well as the intact biosynthetic pathway for the cofactors FAD and CoA. For the biotin biosynthesis pathways, although we did not find genes labeled as *bioH* based on the KEGG annotation results, a homologous gene of *bioH* (Gene ID: AB6T46_01510) was uncovered through additional blastp searches on the *Bartonella* A genome. The gene bears a 39.71% amino acid similarity to *bioH* in *Wolbachia* (Sequence ID: AWV91755.1), making it a promising candidate for replacing *bioH*. Therefore, we anticipate that the biotin biosynthesis pathway is operational in *Bartonella* A. However, *Bartonella* A may retain a limited ability to synthesize other B vitamins due to the absence of some key genes in the other B vitamin biosynthesis pathways, such as thiamine, nicotinate, pantothenate, pyridoxine, and folate.

Considering that some symbiotic bacteria in plant sap–feeding insects play key roles in supplying sufficient quantities of essential amino acids to the host [43], the metabolic reconstruction of essential amino acid biosynthesis pathways was conducted, revealing that *Bartonella* A has a significant deficiency in amino acid synthesis capabilities (Fig. 8C). Of the 11 essential amino acids, only the biosynthesis pathway for the essential amino acid lysine remains intact, indicating that *Bartonella* A has minimal potential to provide the host with essential amino acids.

## Discussion

Arthropods are multi-organismal entities as coined by Prof. Angela E. Douglas [44]. This means that it has become clear that these multicellular organisms are far from being solitary and are commonly occupied by one or multiple microorganisms as integral entities of their existence. Particularly in hematophagous arthropods, it has become clear through extensive research that mosquitoes [45], ticks [46], and tsetse flies [47] harbor symbionts within organs like the midgut and ovary, which are endowed with various biological functions imposed upon processes in host physiology and development. Yet, for *D. gallinae*, a mite primarily infesting birds, research has largely focused on its microbial composition, leaving the functional roles of symbiotic bacteria largely unexplored. Our study delves into (i) the localization of internal bacterial microbiota in all developmental stages of *D. gallinae*, (ii) their internal bacterial diversity, using 16S rRNA amplicon sequencing and conventional culture methods, and (iii) pioneers an investigation into these symbionts’ potential function.

Digestive tract is favored for colonization by multiple microbes in both vertebrates and invertebrates [48, 49]. Similarly, *D. gallinae* hosts a prominent midgut-confined bacterial microbiota, more resembling bacterial tissue-tropism observed in insects [50], and deviating from the observation of bacteria-poor midguts in ticks [51, 52]. The microbial composition of *D. gallinae* was analyzed using conventional culture methods. A variety of bacteria belonging to 20 genera were isolated from *D. gallinae*. While previous studies had identified these genera in *D. gallinae* using methods such as 16S rRNA PCR amplification, TTGE fingerprinting, or amplicon sequencing [4, 5], actual isolation had not been reported.

Then, a more comprehensive analysis of the bacteria in *D. gallinae* was performed by 16S amplicon sequencing in blood-feeding adult females and non-fed individuals. Alpha-diversity indexes of adult fed females were found to be lower than those of eggs and adult males, similar to the pattern previously observed in the *D. gallinae* population from Czechia [15]. The lower bacterial diversity in adult fed females may be attributed to nutritional competition [53]. Adult fed females need to ingest large amounts of blood to maintain their growth and development. After a blood meal, the dominant flora possibly related to blood digestion proliferated rapidly, while inferior flora is suppressed or present at undetectable levels due to the dominance of certain species. Endosymbionts in adult fed females are believed to partially exclude the invasion of other bacteria [29].

The utilization of antibiotics to elucidate the function of symbiotic bacteria in arthropods is a widely used approach. However, this method has not yet been applied to intervene with symbiotic bacteria in *D. gallinae* mites. In ticks, antibiotic treatments targeting endosymbionts cause dysbioses and affected the physiology and development of their hosts [54, 55]. In *Aedes aegypti*, treatment of female mosquitoes with antibiotics inhibits the lysis of RBCs in their intestines and reduced egg production [25]. In this work, the effect of antibiotics on the *D. gallinae* microbiome was analyzed by 16S rRNA gene amplicon sequencing. The results showed a notable decrease in the relative abundance of *Bartonella* A bacteria in the antibiotic-treated group, while the relative abundance of other core flora, such as *Kocuria*, remained largely unaffected. These results were corroborated by qPCR, suggesting that the antibiotic OTC exerted a specific inhibitory effect on the proliferation of *Bartonella* A bacteria. An intriguing observation emerged from the rapid surge in the relative abundance of *Bartonella* A following blood feeding, indicating its plausible involvement in vital life activities of mites subsequent to engorgement. In summary, the adverse effects observed following antibiotic treatment can be attributed to a reduction in the abundance of *Bartonella* A, underscoring the significant role of this bacterium in the blood digestion and reproduction processes of *D. gallinae* mites.

*Bartonella* A, an exclusive symbiotic bacterium identified in *D. gallinae*, is believed to assume a pivotal role in the physiology of *D. gallinae*. *Bartonella* A bacteria were found to be the predominant microbial group within *D. gallinae* from Czechia, constituting ~30% to 70% of the sequences in the *D. gallinae* microbiome, and these bacteria have been observed to exhibit transovarial transmission [4]. Recently, similar findings have been reported in Japanese *D. gallinae* populations. Specifically, that study confirmed the presence of two *Bartonella* species in *D. gallinae*, namely, *Bartonella* sp. group A and *Bartonella* sp. group B. Notably, *Bartonella* sp. group A was detected in all examined mite individuals, suggesting its obligate symbiotic relationship with *D. gallinae* [16]. In the present study, *Bartonella* A represented the second most abundant microbial constituent within the *D. gallinae* microbiome, accounting for 26.75% in eggs, 30.09% in adult females, and 8.95% in adult males. The full-length 16S rRNA gene sequences of this bacterium exhibited a 97.93% similarity to *Bartonella* group A (MF086624) identified in the Czechia population and a 97.28% similarity to *Bartonella* sp. A-YN-2019 (LC710641) found in the Japanese population. In addition, *Bartonella* A had a 100% infection rate among mite individuals in the current study. These findings suggest that the *Bartonella* A bacterium may also function as an obligate symbiont within the Chinese *D. gallinae* population.

It is widely accepted that as bacteria transition into obligate symbionts, their genomes will likely undergo simplification, yet they will continue to possess the capability to produce nutrients such as B vitamins and essential amino acids for their hosts [42, 56]. In the present research, we first isolated *Bartonella* A and completed the construction of its genome, which showed a reduction in genome size and GC content, similar to the patterns observed in other obligate symbionts [57–59]. *Bartonella* A possesses a more streamlined genomic profile in comparison to other *Bartonella* species or strains. Similar genomic characteristics were found in *Rickettsiella* in *D. gallinae*, which is recognized as an obligate symbiont of *D. gallinae* [60]. Nonetheless, it also maintains the basically complete biosynthetic pathways for riboflavin, biotin, and the intact biosynthetic pathway of the cofactors FAD and CoA. In contrast, the biosynthesis pathways for thiamine, nicotinate, pantothenate, pyridoxine, and folate seem to be less cohesive, and it is uncertain whether these pathways are actually functioning. With the retention of a long biotin biosynthesis pathway in the *Bartonella* A genome (8/9 genes present) and the presence of homologous genes of *bioH*, it is likely that the biotin biosynthesis pathway is functional in *Bartonella* A. Additionally, the metabolic reconstruction of essential amino acid biosynthesis pathways revealed that, apart from lysine, the biosynthetic pathways for the other essential amino acids have varying degrees of absence. Since *D. gallinae* feeds on blood and is capable of breaking down hemoglobin and other blood proteins to release free amino acids [61], it is probable that the mite has an abundance of essential and non-essential amino acids that fulfill its nitrogen needs. Therefore, *Bartonella* A might not play a key role in supplying essential amino acids to the host. Furthermore, we conducted a reclassification of *Bartonella* A based on the results of the phylogenetic tree, ANI, and DDH value, finding it is a new species within the genus *Bartonella*, which was named as *Bartonella kongi* sp. nov. To our knowledge, it is the first time that an obligate symbiont has been isolated and identified from *D. gallinae*, which has been further identified as a new species of *Bartonella*. Further mechanisms of *Bartonella kongi* sp. nov. playing roles in the fitness of *D. gallinae* are being conducted in our laboratory.

## Conclusion

In summary, our investigation unveils details on the symbiotic relationship between *D. gallinae* mites and their bacterial microbiota. Our findings not only reveal the prominence of midgut-confinement bacterial communities, with the *Kocuria* and *Bartonella* A (a new species, later identified as *Bartonella kongi* sp. nov.) as predominant inhabitants, but also highlight the dynamic changes in bacterial abundance post-blood ingestion, indicative of their significant involvement in post-engorgement activities. Furthermore, the isolation and characterization of bacterial strains exhibiting hemolytic activities underscore their potential contribution to blood digestion within the mites. Importantly, our study demonstrates the functional significance of these symbiotic bacteria through antibiotic-induced dysbiosis experiments, revealing profound impacts on mite physiology, including delayed blood digestion, reproductive impairments, and compromised embryo development. Our investigation also revealed *Bartonella* A as a new species of the genus *Bartonella*, which was identified as an obligate symbiont with the potential to supply biotin to *D. gallinae*. By combining molecular, microbiological, and phenotypic analyses, our study contributes to a deeper understanding of the complex interactions between arthropod hosts and their endosymbiotic bacteria. These results underscore the essential role of microbiota in the biology of poultry red mites and suggest microbiota-targeted interventions as potential strategies for controlling mite populations on poultry farms. Future research in this area holds promise for the development of novel and practical approaches for managing ectoparasite infestations in agricultural settings, ultimately benefiting both animal welfare and farm productivity.

## Supplementary Material

Supplementary_file_1_ycae127

Supplementary_file_2_ycae127

## Data Availability

All data generated or analyzed during this study are included in this published article and its Additional file. All of the original sequences obtained in this work have been deposited in the National Center for Biotechnology Information (NCBI) under project number PRJNA944571, PRJNA1079531, and PRJNA1142099.
