# Correction to: Significant role of symbiotic bacteria in the blood digestion and reproduction of *Dermanyssus gallinae* mites

**DOI:** 10.1093/ismeco/ycae166

**Published:** 2025-04-15

This is a correction to: Qi Liu, Tiancong Sun, Penglong Wang, Lifang Wang, Helena Frantova, David Hartmann, Jan Perner, Weiwei Sun, Baoliang Pan, Significant role of symbiotic bacteria in the blood digestion and reproduction of *Dermanyssus gallinae* mites, *ISME Communications*, Volume 4, Issue 1, January 2024, ycae127, https://doi.org/10.1093/ismeco/ycae127

In the originally published version, a third supplementary file was omitted. The file has now been added.

