# Supplementary material for: Significant role of symbiotic bacteria in the blood digestion and reproduction of Dermanyssus gallinae mites
Source: ISME Commun. 2024 Oct 30;4(1):ycae127. doi: 10.1093/ismeco/ycae127 (PMC11550332; doi:10.1093/ismeco/ycae127)
Supplement: Supplementary_file_1_ycae127 [file supplementary_file_1_ycae127.pdf]

Table S1 The primers used in this study.

Group	Name	Primer (5'-3')	Size (bp)
Universal, 16S rRNA, PCR Pacbio	27F	AGRGTTYGATYMTGGCTCAG	1 500
	1492R	RGYTACCTTGTTACGACTT	
Universal, 16S rRNA, qPCR	Com1	CAGCAGCCGCGGTAATAC	270
	769R	ATCCTGTTTGMTMCCCVCRC	
Universal ,16S rRNA, PCR Illumina	338F	ACTCCTACGGGAGGCAGCAG	468
	806R	GGACTACHVGGGTWTCTAAT	
un-Rhi (*Bartonella* A) 16S rRNA, PCR	Bar1-F	GGGGGAAAGATTTATCGGATT	800
	Bar1-R	TGAAGAAATCTATCTCTAAATCTCA	
un-Rhi (*Bartonella* A) 16S rRNA, qPCR	Bar2-F	GAAGCTAGCCGTTGGAAGTT	181
	Bar2-R	TGAAGAAATCTATCTCTAAATCTCA	
Kocuria 16S rRNA, PCR	Koc1-F	CCACACTGGGACTGAGACAC	709
	Koc1-R	AGCCCCGAAAGGAAACAACA	
Kocuria 16S rRNA, qPCR	Koc2-F	CTTATCCCAGAGTCCAAGGT	134
	Koc2-R	ATGGCTCAGGACGAACGCTG	

Table S5 Summary of Sequence and Base Counts for 16S rRNA Amplicon Sequencing

Sample	unfiltered reads number	Base number	Rarefied reads number
E1	48045	19624292	43749
E2	54282	22234827	43749
E3	54981	22343440	43749
AF1 (EA1)	60111	24500236	43749
AF2 (EA2)	54307	22133958	43749
AF3 (EA3)	61513	25094155	43749
AM1	71847	30347532	43749
AM2	64554	26483455	43749
AM3	60891	24866034	43749
SA1	63457	26416098	43749
SA2	58470	24100993	43749
SA3	61975	25355855	43749
C1	58542	23722967	43749
C2	58819	24001891	43749
C3	57769	23562187	43749
T1	53273	22101656	43749
T2	46312	19057626	43749
T3	49126	20174737	43749

Table S6 Hemolytic activity of isolated bacteria from *D. gallinae*

Bacterial genus	The width of the lysis zones (mm)	Hemolysis type
Bacillus sp.	3.74±0.26	β
Clostridium sp.	4.05±0.16	β
Pseudomonas sp.	1.69±0.12	α
Enterococcus sp.	1.06±0.08	α
Proteus sp.	7.54±0.36	β

Table S7 Recovery of reproductive capacity of adult female mites after antibiotic treatment

Reproduction parameters	First blood feeding (With antibiotics)	Second blood feeding (No antibiotics)	Third blood feeding (No antibiotics)	Fourth blood feeding (No antibiotics)
Oviposition rate (%)	64.93±20.08^{a}	92.62±0.86^{ab}	96.66±1.52^{b}	97.22±1.46^{b}
Fecundity	1.05±0.4^{a}	2.23±0.12^{b}	3.86±0.17^{c}	4.28±0.24^{cd}
Hatching rate (%)	22.54±13.19^{a}	69.91±3.45^{b}	96.28±1.96^{c}	95.72±2.00^{cd}

The data are expressed as the mean ± SD

a, b, c, d Values within the same row followed by different letters are significantly different

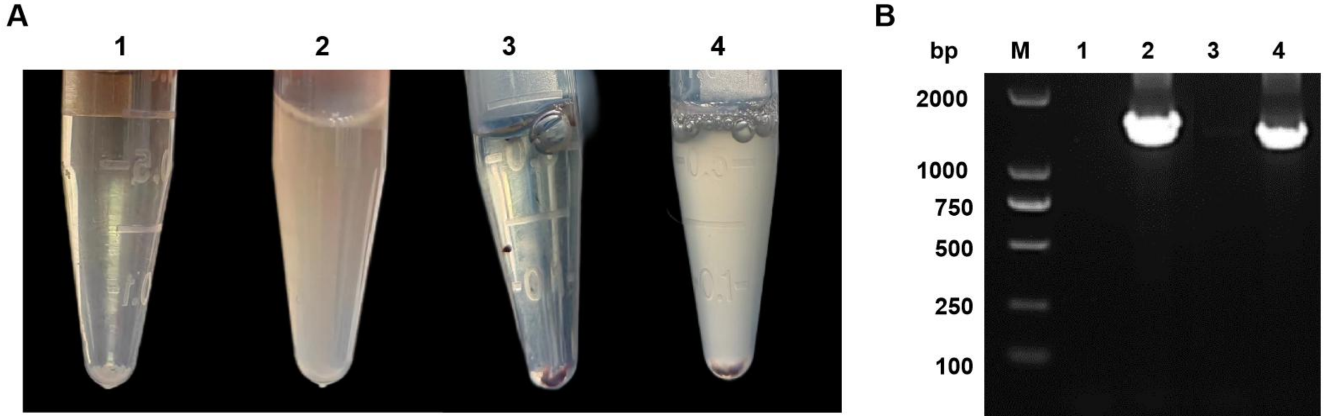

Fig. S1 Evaluation of surface sterilization efficiency *D. gallinae* mites and their eggs. A Photo images of LB media after external washing mites or eggs and subsequent thermal culturing. 1: surface-cleaned eggs; 2: no treatment eggs; 3: surface-cleaned mites; 4: no treatment mites. **B** PCR amplification of the bacterial 16S rRNA genes from the corresponding media in Fig A.

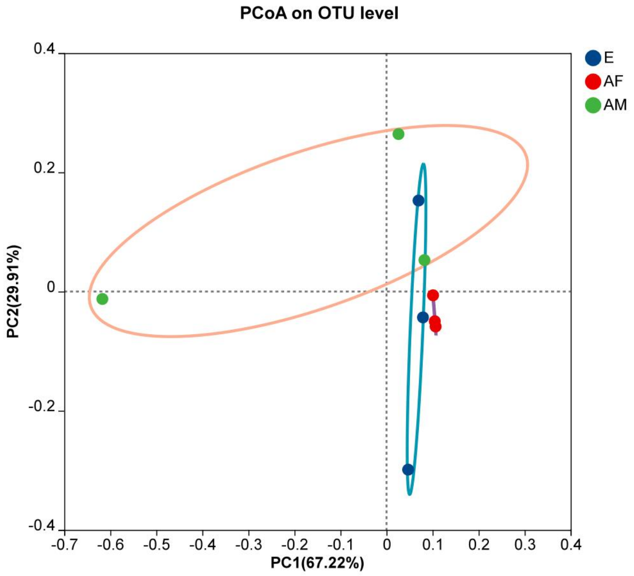

Fig. S2 The PCoA analysis for bacterial community composition in based on Bray-Curtis. Distances between points on the ordination plot reflect relative dissimilarities in microbiome structures. E, AF, and AM indicated the egg, fed adult female, and adult male, respectively.

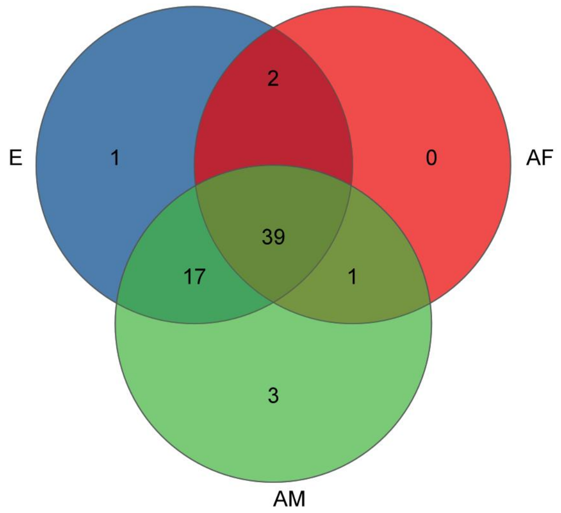

Fig. S3 Venn diagram representing the distribution of the OTUs across different groups of *D. gallinae*. Shared bacteria OTUs are shown in core. E, AF, and AM indicated the eggs, fed adult females, and adult males, respectively.

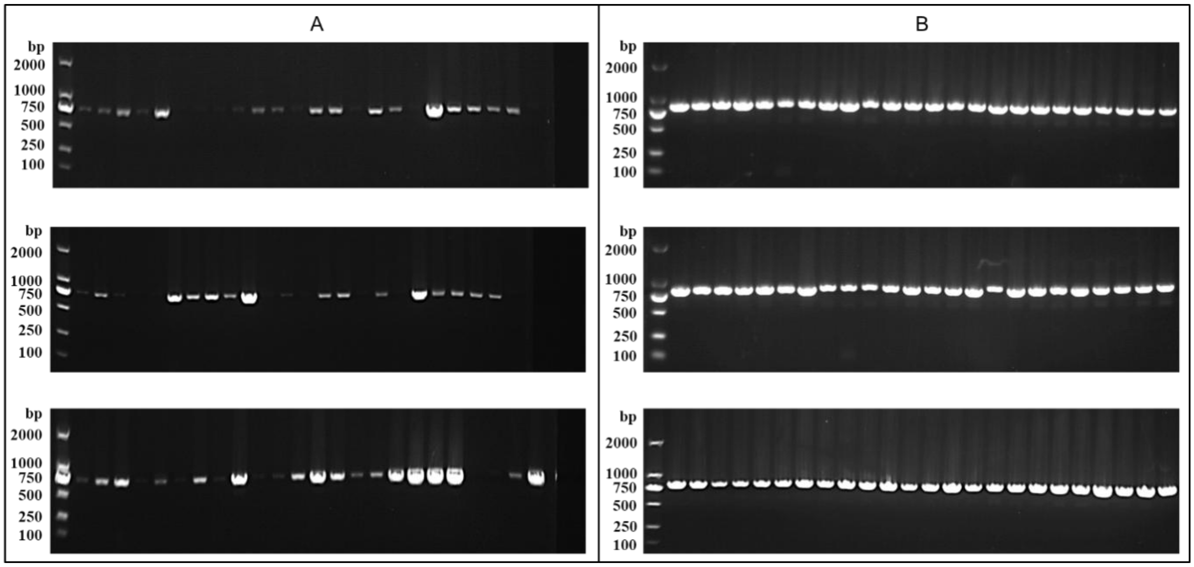

Fig. S4 Detection of infection rates of core bacteria in individual adult female mites by specific PCR amplification. **A** *Kocuria*; **B** *Bartonella* A.

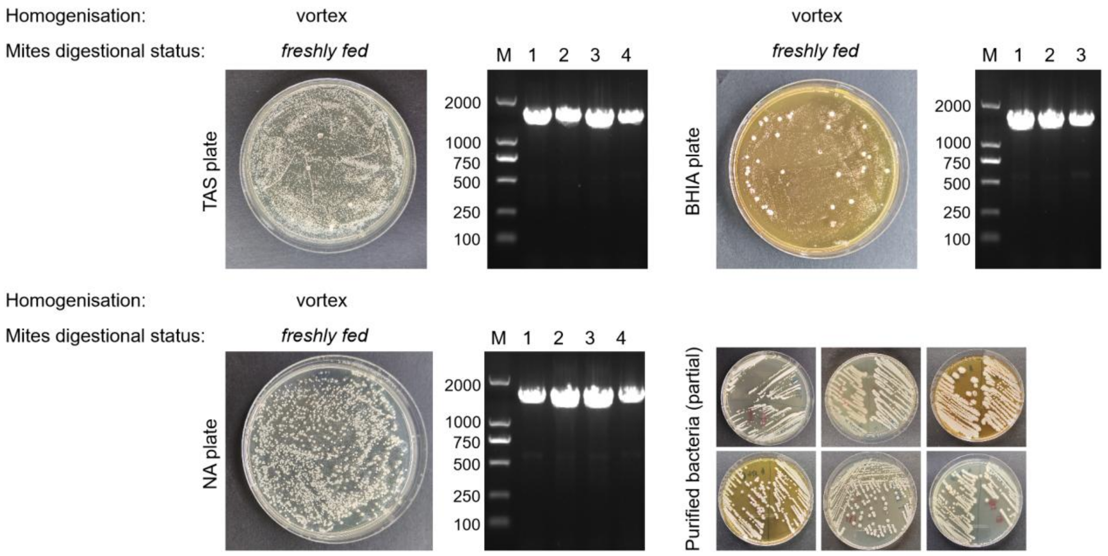

Fig. S5. Agar cultivation and 16S rRNA gene amplification of bacteria isolated from dissected midguts of *D. gallinae* mites. A homogenisation procedure was applied on midguts of freshly fed *D. gallinae* adult females. Samples from different plates were taken for DNA isolation and 16S rRNA amplification with subsequent Sanger sequencing. Individual sequences are shown below as Suplementary Data S1.

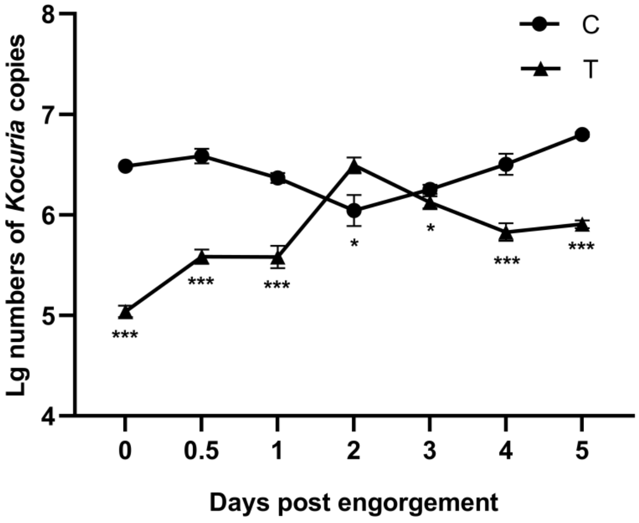

Fig. S6 The effect of antibiotics treatment on *Kocuria* abundance in *D. gallinae*. The quantification of numbers of copies of *Kocuria* 16S rRNA gene in control and treatment groups. The data are Log10 transformed. Asterisks indicate the statistical significance: *p-value < 0.05; **p-value < 0.01; ***p-value < 0.001 in the Students't test.

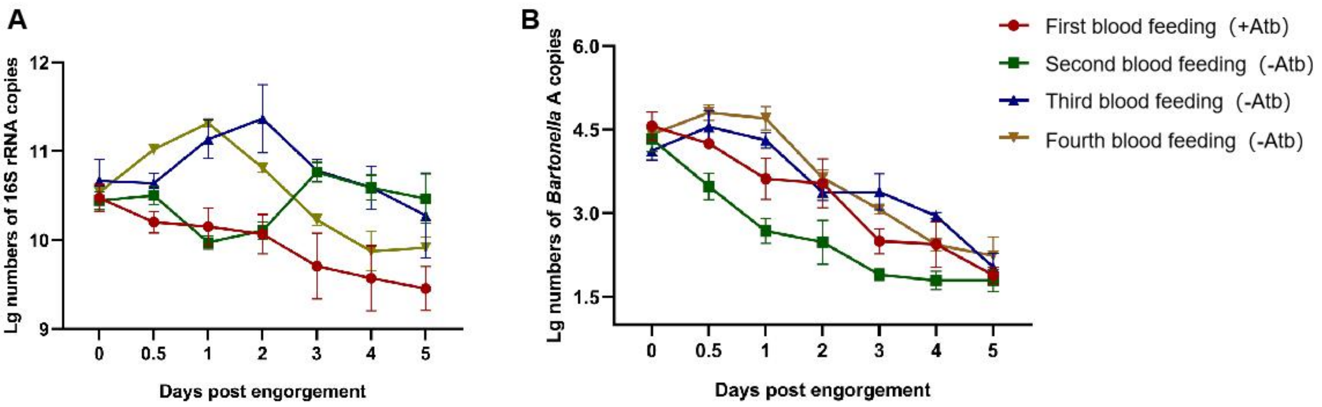

Fig. S7. Recovery of bacterial load of adult female mites after antibiotic treatment. The quantification of numbers of copies of 16S rRNA gene in different groups. The data are Log_{10} transformed. **A** Numbers of copies obtained from adult females by universal primers. **B** Numbers of copies obtained from adult females by *Bartonella* A specific primers. +Atb: The mites fed on OTC-treated chicken. -Atb: The mites fed on non-treated chicken.

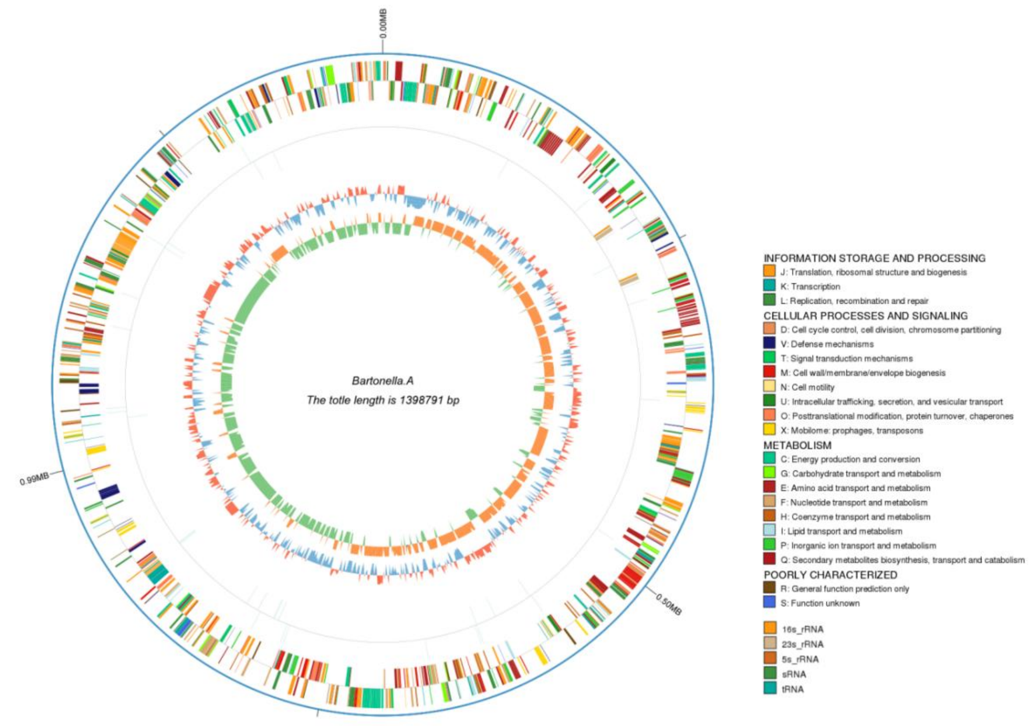

Fig. S8. Circular genome representation map of the *Bartonella* A symbiont. From outside to the centre: genome sequence of coordinates, genes on forward strand (colour by COG categories), genes on reverse strand (colour by COG categories), ncRNA, GC content, GC skew.

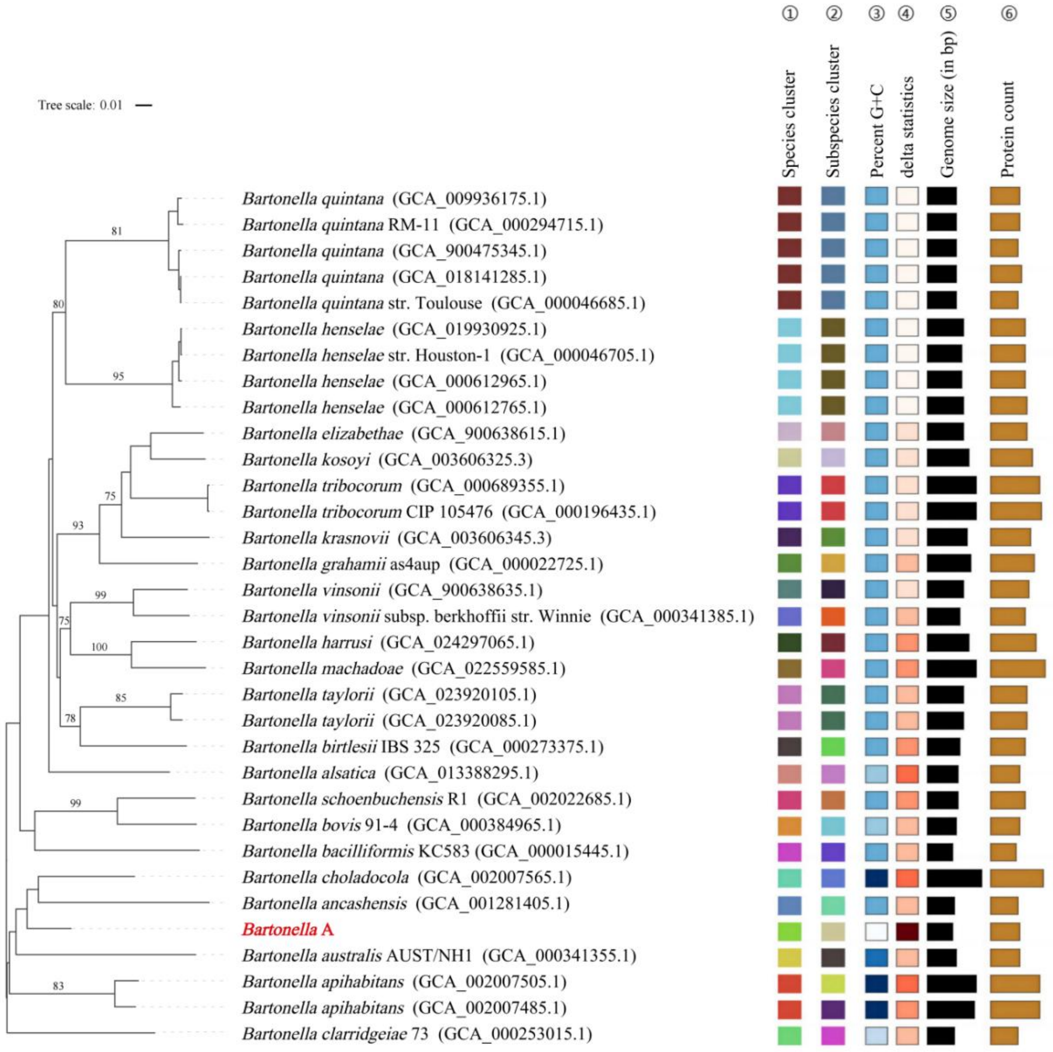

Fig. S9. Whole-genome-based phylogeny. The resulting intergenomic distances were used to infer a balanced minimum evolution tree with branch support via FASTME 2.1.6.1. Branch support was inferred from 100 pseudo-bootstrap replicates each, and values > 75% are shown above branches. Leaf labels are annotated by affiliation to species ① and subspecies ② clusters, genomic G+C content ③, δ

values ④, overall genome sequence length ⑤, number of proteins ⑥. Numbers in brackets indicate NCBI accession numbers.

Supplementary Data S1. Nucleotide sequences of 16S rRNA amplicons acquired from agar plates culturing and electrophoretic separation above.

>TSA1 *Staphyloccocus*

TAAACAGTGGGGCCGGCTTAATACATGCAAGTCGAGCGAACAGATAAGGAGCTTGCTCCTTT
GACGTTAGCGGCGGAAGGGTGAGTAACACGTGGGTAACCTACCTATAAGACTGGGATAACTT
CGGGAAACCGGAGCTAATACCGGATAACATTTGGAACCGCATGGTTCTAAAGTGAAAGATGG
TTTTGCTATCACTTATAGATGGACCCGCGCCGTATTAGCTAGTTGGTAAGGTAACGGCTTACC
AAGGCGACGATACGTAGCCGACCTGAGAGGGTGATCGGCCACACTGGAACTGAGACACGGT
CCAGACTCCTACGGGAGGCAGCAGTAGGGAATCTTCCGCAATGGGCGAAAGCCTGACGGAG
CAACGCCGCGTGAGTGATGAAGGGTTTCGGCTCGTAAAACTCTGTTATTAGGGAAGAACAA
ATGTGTAAGTAACTGTGCACATCTTGACGGTACCTAATCAGAAAGCCACGGCTAACTACGTG
CCAGCAGCCGCGGTAATACGTAGGTGGCAAGCGTTATCCGGAATTATTGGGCGTAAAGCGCG
CGTAGGCGGTTTCTTAAGTCTGATGTGAAAGCCCACGGCTCAACCGTGGAGGGTCATTGGAA
ACTGGGAAACTTGAGTGCAGAAGAGGAAAGTGGAATTCCATGTGTAGCGGTGAAATGCGCA
GAGATATGGAGGAACACCAGTGGCGAAGGCGACTTTCTGGTCTGTAACTGACGCTGATGTG
CGAAAGCGTGGGGATCAAACAGGATTAGATACCCTGGTAGTCCACGCCGTAAACGATGAGT
GCTAAGTGTTAGGGGGTTTCCGCCCCTTAGTGCTGCAGCTAACGCATTAAGCACTCCGCCTG
GGGAGTACGACCGCAAGGTTGAAACTCAAAGGAATTGACGGGGACCCGCACAAGCGGTGG
AGCATGTGGTTTAATTCGAAGCAACGCGAAGAACCTTACCAAATCTTGACATCCTTTGAAAA
CTCTAGAGATAGAGCCTTCCCCTTCGGGGGACAAAGTGACAGGTGGTGCATGGTTGTCGTCA
GCTCGTGTCGTGAGATGTTGGGTTAAGTCCCGCAACGAGCGCAACCCTTAAGCTTAGTTGCC
ATCATTAAGTTGGGCACTCTAAGTTGACTGCCGGTGACAAACCGGAGGAAGGTGGGGATGA
CGTCAAATCATCATGCCCCTTATGATTTGGGCTACACACGTGCTACAATGGACAATACAAAGG

GCAGCTAAACCGCGAGGTCATGCAAATCCCATAAAGTTGTTCTCAGTTCGGATTGTAGTCTG

CAACTCGACTACATGAAGCTGGAATCGCTAGTAATCGTAGATCAGCATGCTACGGTGAATACG

TTCCCGGGTCTTGTACACACCGCCCGTCACCCCACGAGAGTTTGTAACACCCGAAGCCGGTG

GAGTAACCATTTATGGAGCTAGCCGTCGAAAGGGTGAACCAAAGGGG

>TSA2 *Staphyloccocus*

GCACCTGGGCGGCGTGCCTATACATGCAAGTCGAGCGAACAGATAAGGAGCTTGCTCCTTTG

ACGTTAGCGGCGGACGGGTGAGTAACACGTGGGTAACCTACCTATAAGACTGGGATAACTTC

GGGAAACCGGAGCTAATACCGGATAACATTTGGAACCGCATGGTTCTAAAGTGAAAGATGGT

TTTGCTATCACTTATAGATGGACCCGCGCCGTATTAGCTAGTTGGTAAGGTAACGGCTTACCA

AGGCGACGATACGTAGCCGACCTGAGAGGGTGATCGGCCACACTGGAACTGAGACACGGTC

CAGACTCCTACGGGAGGCAGCAGTAGGGAATCTTCCGCAATGGGCGAAAGCCTGACGGAGC

AACGCCGCGTGAGTGATGAAGGGTTTCGGCTCGTAAAACTCTGTTATTAGGGAAGAACAAAT

GTGTAAGTAACTGTGCACATCTTGACGGTACCTAATCAGAAAGCCACGGCTAACTACGTGCC

AGCAGCCGCGGTaATACGTAGGTGGCAAGCGtTATCCGGAATTATTGGGCGTAAAGCGCGCGT

AGGCGGTTTCTTAAGTCTGATGTGAAAGCCCACGGCTCAACCGTGGAGGGTCATTGGAAAC

TGGGAAACTTGAGTGCAGAAGAGGAAAGTGGAATTCCATGTGTAGCGGTGAAATGCGCAGA

GATATGGAGGAACACCAGTGGCGAAGGCGACTTTCTGGTCTGTAACTGACGCTGATGTGCG

AAAGCGTGGGGATCAAACAGGATTAGATACCCTGGTAGTCCACGCCGTAAACGATGAGTGCT

AAGTGTTAGGGGGTTTCCGCCCCTTAGTGCTGCAGCTAACGCATTAAGCACTCCGCCTGGGG

AGTACGACCGCAAGGTTGAAACTCaAAGGAATTGACGGGGACCCGCACAAGCGGTGGAGCA

TGTGGTTTAATTCGAAGCAACGCGaAGAACCTTACCAAATCTTGACATCCTTTGaAAACTCTA

GAGATAGAGCCTTCCCCTTCGGGGGACAAAGTGACAGGTGGTGCATGGTTGTCGTCAGCTC

GTGTCGTGAGATGTTGGGTTAAGTCCCGCAACGAGCGCAACCCTTAAGCTTAGTTGCCATCA

TTAAGTTGGGCACTCTAGGTTGACTGCCGGTGACAAACCGGAGGAAGGTGGGGATGACGTC

AAATCATCATGCCCCTTATGATTTGGGCTACACACGTGCTACAATGGACAATACAAAGGGCAG

CTAAACCGCGAGGTCATGCAAATCCCATAAAGTTGTTCTCAGTTCGGATTGTAGTCTGCAACT

CGACTACATGAAGCTGGAATCGCTAGTAATCGTAGATCAGCATGCTACGGTGAATACGTTCCC

GGGTCTTGTACACACCGCCCGTCACACCACGAGAGTTTGTAACACCCGAAGCCGGTGGAGT
AACCATTTATGGAGCTAGCCCGTCGAAAGGGTGGACCCAAGATTG

>TSA3 *Staphyloccocus*

AGGCACAGTGGCGGCCGGTGCCTAATACATGCAAGTCGAGCGAACAGATAAGGAGCTTGCT
CCTTTGACGTTAGCGGCGGAAGGGTGAGTAACACGTGGGTAACCTACCTATAAGACTGGGAT
AACTTCGGGAAACCGGAGCTAATACCGGATAACATTTGGAACCGCATGGTTCTAAAGTGAAA
GATGGTTTTGCTATCACTTATAGATGGACCCGCGCCGTATTAGCTAGTTGGTAAGGTAACGGC
TTACCAAGGCGACGATACGTAGCCGACCTGAGAGGGTGATCGGCCACACTGGAACTGAGAC
ACGGTCCAGACTCCTACGGGAGGCAGCAGTAGGGAATCTTCCGCAATGGGCGAAAGCCTGA
CGGAGCAACGCCGCGTGAGTGATGAAGGGTTTCGGCTCGTAAAACTCTGTTATTAGGGAAG
AACAAATGTGTAAGTAACTGTGCACATCTTGACGGTACCTAATCAGAAAGCCACGGCTAACT
ACGTGCCAGCAGCCGCGGTAATACGTAGGTGGCAAGCGTTATCCGGAATTATTGGGCGTAAA
GCGCGCGTAGGCGGTTTCTTAAGTCTGATGTGAAAGCCCACGGCTCAACCGTGGAGGGTCAT
TGGAAACTGGGAAACTTGAGTGCAGAAGAGGAAAGTGGAATTCCATGTGTAGCGGTGAAAT
GCGCAGAGATATGGAGGAACACCAGTGGCGAAGGCGACTTTCTGGTCTGTAACTGACGCTG
ATGTGCGAAAGCGTGGGGATCAAACAGGATTAGATACCCTGGTAGTCCACGCCGTAAACGAT
GAGTGCTAAGTGTTAGGGGGTTTCCGCCCCTTAGTGCTGCAGCTAACGCATTAAGCACTCCG
CCTGGGGAGTACGACCGCAAGGTTGAAACTCAAAGGAATTGACGGGGACCCGCACAAGCG
GTGGAGCATGTGGTTTAATTCGAAGCAACGCGAAGAACCTTACCAAATCTTGACATCCTTTG
AAAACTCTAGAGATAGAGCCTTCCCCTTCGGGGGACAAAGTGACAGGTGGTGCATGGTTGT
CGTCAGCTCGTGTCGTGAGATGTTGGGTTAAGTCCCGCAACGAGCGCAACCCTTAAGCTTAG
TTGCCATCATTAAGTTGGGCACTCTAAGTTGACTGCCGGTGACAAACCGGAGGAAGGTGGG
GATGACGTCAAATCATCATGCCCCTTATGATTTGGGCTACACACGTGCTACAATGGACAATAC
AAAGGGCAGCTAAACCGCGAGGTCATGCAAATCCCATAAAGTTGTTCTCAGTTCGGATTGTA
GTCTGCAACTCGACTACATGAAGCTGGAATCGCTAGTAATCGTAGATCAGCATGCTACGGTG
AATACGTTCCCGGGTCTTGTACACACCGCCCGTCACCCCACGAGAGTTTGTAACACCCGAAG
CCGGTGGAGTAACCATTTATGGAGCTAGCCGTCGAAAGGTGGAACCAAAAATTT

>TSA4 *Koucria*

GGCACGTGGCGGCGTGCTTACCATGCAAGTCGAACGCTGAAGCTTGGTGCTTGCACCGGGT

GGATGAGTGGCGAACGGGTGAGTAATACGTGAGTGACCTACCTTGGACTCTGGGATAAGCCT

GGGAAACTGGGTCTAATACCGGATATGACCAATGGGCGCATGCTCTGTTGGTGGAAAGGGTT

TGTACTGGTCTTTGATGGGCTCACGGCCTATCAGCTTGTTGGTGAGGTAATGGCTTACCAAGG

CGACGACGGGTAGCCGGCCTGAGAGGGTGACCGGCCACACTGGGACTGAGACACGGCCCA

GACTCCTACGGGAGGCAGCAGTGGGGAATATTGCACAATGGGCGCAAGCCTGATGCAGCGA

CGCCGCGTGAGGGATGACGGCCTTCGGGTTGTAAACCTCTTTCAGCAGGGAAGAAGCGAAA

GTGACGGTACCTGCAGAAGAAGCGCCGGCTAACTACGTGCCAGCAGCCGCGGTAATACGTA

GGGCGCAAGCGTTGTCCGGAATTATTGGGCGTAAAGAGCTCGTAGGCGGTTTGTCGCGTCTG

CTGTGAAAGCCCGGGGCTTAACCCCGGGTGTGCAGTGGGTACGGGCAGGCTAGAGTGCAGT

AGGGGAGACTGGAATTCCTGGTGTAGCGGTGAAATGCGCAGATATCAGGAGGAACACCGAT

GGCGAAGGCAGGTCTCTGGGCTGTTACTGACGCTGAGGAGCGAAAGCATGGGGAGCGAAC

AGGATTAGATACCCTGGTAGTCCATGCCGTAAACGTTGGGCACTAGGTGTGGGGGACATTCC

ACGTTTTCCGCGCCGTAGCTAACGCATTAAGTGCCCCGCCTGGGGAGTACGGCCGCAAGGCT

AAAACTCAAAGGAATTGACGGGGGCCCGCACAAGCGGCGGAGCATGCGGATTAATTCGATG

CAACGCGAAGAACCTTACCAAGGCTTGACATACACCGGATCGCAGCAGAGATGTTGTTTCCT

TTCGGGGCTGGTGTACAGGTGGTGCATGGTTGTCGTCAGCTCGTGTCGTGAGATGTTGGGTT

AAGTCCCGCAACGAGCGCAACCCTCGTTCTATGTTGCCAGCACGTGATGGTGGGGACTCATA

GGAGACTGCCGGGGTCAACTCGGAGGAAGGTGGGGATGACGTCAAATCATCATGCCCCTTA

TGTCTTGGGCTTCACGCATGCTACAATGGCCGGTACAATGGGTTGCGATACTGTGAGGTGGA

GCTAATCCCAAAAAGCCGGTCTCAGTTCGGATTGGGGTCTGCAACTCGACCCCATGAAGTCG

GAGTCGCTAGTAATCGCAGATCAGCAACGCTGCGGTGAATACGTTCCCGGGCCTTGTACACA

CCGCCCGTCAAGTCACGAAAGTCGGTAACACCCGAAGCCGATGGCCTAACCCTTTGTGGGG

GGAGTCGTCGAAAGTTGACCGGTCCT

>NA1 *Escherichia*

CAATTGGCGGCAGGCCTACACATGCAAGTCGAACGGTAACAGGAAGCAGCTTGCTTCTTTG

CTGACGAGTGGCGGACGGGTGAGTAATGTCTGGGAAACTGCCTGATGGAGGGGGATAACTA
CTGGAAACGGTAGCTAATACCGCATAACGTCGCAAGACCAAAGAGGGGGACCTTCGGGCCT
CTTGCCATCGGATGTGCCCAGATGGGATTAGCTAGTAGGTGGGGTAACGGCTCACCTAGGCG
ACGATCCCTAGCTGGTCTGAGAGGATGACCAGCCACACTGGAACTGAGACACGGTCCAGAC
TCCTACGGGAGGCAGCAGTGGGGAATATTGCACAATGGGCGCAAGCCTGATGCAGCCATGC
CGCGTGTATGAAGAAGGCCTTCGGGTTGTAAAGTACTTTCAGCGGGGAGGAAGGGAGTAAA
GTTAATACCTTTGCTCATTGACGTTACCCGCAGAAGAAGCACCGGCTAACTCCGTGCCAGCA
GCCGCGGTAATACGGAGGGTGCAAGCGTTAATCGGAATTACTGGGCGTAAAGCGCACGCAG
GCGGTTTGTTAAGTCAGATGTGAAATCCCCGGGCTCAACCTGGGAACTGCATCTGATACTGG
CAAGCTTGAGTCTCGTAGAGGGGGGTAGAATTCCAGGTGTAGCGGTGAAATGCGTAGAGAT
CTGGAGGAATACCGGTGGCGAAGGCGGCCCCCTGGACGAAGACTGACGCTCAGGTGCGAA
AGCGTGGGGAGCAAACAGGATTAGATACCCTGGTAGTCCACGCCGTAAACGATGTCGACTTG
GAGGTTGTGCCCTTGAGGCGTGGCTTCCGGAGCTAACGCGTTAAGTCGACCGCCTGGGGAG
TACGGCCGCAAGGTTAAAACTCAAATGAATTGACGGGGGCCCGCACAAGCGGTGGAGCATG
TGGTTTAATTCGATGCAACGCGAAGAACCTTACCTGGTCTTGACATCCACGGAAGTTTTCAG
AGATGAGAAGGTGCCTTCGGGAACCGTGAGACAGGTGCTGCATGGCTGTCGTCAGCTCGTG
TTGTGAAATGTTGGGTTAAGTCCCGCAACGAGCGCAACCCTTATCCTTTGTTGCCAGCGGTC
CGGCCGGGAACTCAAAGGAGACTGCCAGTGATAAACTGGAGGAAGGTGGGGATGACGTCA
AGTCATCATGGCCCTTACGACCAGGGCTACACACGTGCTACAATGGCGCATACAAAGAGAAG
CGACCTCGCGAGAGCAAGCGGACCTCATAAAGTGCGTCGTAGTCCGGATTGGAGTCTGCAA
CTCGACTCCATGAAGTCGGAATCGCTAGTAATCGTGGATCAGAATGCCACGGTGAATACGTT
CCCGGGCCTTGTACACACCGCCCGTCACACCATGGGAGTGGGTTGCAAAAGAAGTAGGTAG
CTTAACCTTCGGGAGGGCGCCTACCAACTTGGATTTAATGGGGGGGTG

>NA2 *Staphyloccocus*

AAACGTGCCGGGGGGCTAATACATGCAAGTCGAGCGAACAGATGAGAAGCTTGCTTCTCTG
ATGTTAGCGGCGGACGGGTGAGTAACACGTGGGTAACCTACCTATAAGACTGGGATAACTCC
GGGAAACCGGGGCTAATACCGGATAATATATTGAACCGCATGGTTCAATGTTGAAAGACGGT

TTCGGCTGTCACTTATAGATGGACCCGCGCCGTATTAGCTAGTTGGTAAGGTAACGGCTTACC
AAGGCAACGATACGTAGCCGACCTGAGAGGGTGATCGGCCACACTGGAACTGAGACACGGT
CCAGACTCCTACGGGAGGCAGCAGTAGGGAATCTTCCGCAATGGGCGAAAGCCTGACGGAG
CAACGCCGCGTGAGTGATGAAGGTCTTAGGATCGTAAAACTCTGTTGTTAGGGAAGAACAA
ATTTGTTAGTAACTGAACAAGTCTTGACGGTACCTAACCAGAAAGCCACGGCTAACTACGTG
CCAGCAGCCGCGGTAATACGTAGGTGGCAAGCGTTATCCGGAATTATTGGGCGTAAAGCGCG
CGTAGGCGGTTTCTTAAGTCTGATGTGAAAGCCCACGGCTCAACCGTGGAGGGTCATTGGAA
ACTGGGGAACTTGAGTGCAGAAGAGGAGAGTGGAATTCCATGTGTAGCGGTGAAATGCGCA
GAGATATGGAGGAACACCAGTGGCGAAGGCGGCTCTCTGGTCTGTAACTGACGCTGATGTG
CGAAAGCGTGGGGATCAAACAGGATTAGATACCCTGGTAGTCCACGCCGTAAACGATGAGT
GCTAAGTGTTAGGGGGTTTCCGCCCCTTAGTGCTGCAGCTAACGCATTAAGCACTCCGCCTG
GGGAGTACGACCGCAAGGTTGAAACTCAAAGGAATTGACGGGGACCCGCACAAGCGGTGG
AGCATGTGGTTTAATTCGAAGCAACGCGAAGAACCTTACCAAATCTTGACATCCTTTGATCG
CTCTAGAGATAGAGTTTTCCCCTTCGGGGGACAAAGTGACAGGTGGTGCATGGTTGTCGTCA
GCTCGTGTCGTGAGATGTTGGGTTAAGTCCCGCAACGAGCGCAACCCTTAAGCTTAGTTGCC
ATCATTAAGTTGGGCACTCTAGGTTGACTGCCGGTGACAAACCGGAGGAAGGTGGGGATGA
CGTCAAATCATCATGCCCCTTATGATTTGGGCTACACACGTGCTACAATGGATAATACAAAGG
GCAGCGAACCCGCGAGGTCAAGCAAATCCCATAAAATTATTCTCAGTTCGGATTGTAGTCTG
CAACTCGACTACATGAAGCTGGAATCGCTAGTAATCGTAGATCAGCATGCTACGGTGAATACG
TTCCCGGGTCTTGTACACACCGCCCGTCACACCACGAGAGTTTGTAACACCCGAAGCCGGT
GGAGTAACCTTTTATTAGGAGCTAGCCGTCGAAGGGTGAACCGGGATGT

>NA3 *Staphyloccocus*

CAAACAGTGGCGGCGGTGCCTATAATGCAAGTCGAGCGAACAGATGAGAAGCTTGCTTCTC
TGATGTTAGCGGCGGACGGGTGAGTAACACGTGGGTAACCTACCTATAAGACTGGGATAACT
CCGGGAAACCGGGGCTAATACCGGATAATATATTGAACCGCATGGTTCAATGTTGAAAGACG
GTTTCGGCTGTCACTTATAGATGGACCCGCGCCGTATTAGCTAGTTGGTAAGGTAACGGCTTA
CCAAGGCAACGATACGTAGCCGACCTGAGAGGGTGATCGGCCACACTGGAACTGAGACACG

GTCCAGACTCCTACGGGAGGCAGCAGTAGGGAATCTTCCGCAATGGGCGAAAGCCTGACGG

AGCAACGCCGCGTGAGTGATGAAGGTCTTAGGATCGTAAAACTCTGTTGTTAGGGAAGAAC

AAATTTGTTAGTAACTGAACAAGTCTTGACGGTACCTAACCAGAAAGCCACGGCTAACTACG

TGCCAGCAGCCGCGGTAATACGTAGGTGGCAAGCGTTATCCGGAATTATTGGGCGTAAAGCG

CGCGTAGGCGGTTTCTTAAGTCTGATGTGAAAGCCCACGGCTCAACCGTGGAGGGTCATTGG

AAACTGGGGAACTTGAGTGCAGAAGAGGAGAGTGGAATTCCATGTGTAGCGGTGAAATGCG

CAGAGATATGGAGGAACACCAGTGGCGAAGGCGGCTCTCTGGTCTGTAACTGACGCTGATG

TGCGAAAGCGTGGGGATCAAACAGGATTAGATACCCTGGTAGTCCACGCCGTAAACGATGA

GTGCTAAGTGTTAGGGGGTTTCCGCCCCTTAGTGCTGCAGCTAACGCATTAAGCACTCCGCC

TGGGGAGTACGACCGCAAGGTTGAAACTCAAAGGAATTGACGGGGACCCGCACAAGCGGT

GGAGCATGTGGTTTAATTCGAAGCAACGCGAAGAACCTTACCAAATCTTGACATCCTTTGAT

CGCTCTAGAGATAGAGTTTTCCCCTTCGGGGGACAAAGTGACAGGTGGTGCATGGTTGTCGT

CAGCTCGTGTCGTGAGATGTTGGGTTAAGTCCCGCAACGAGCGCAACCCTTAAGCTTAGTTG

CCATCATTAAGTTGGGCACTCTAGGTTGACTGCCGGTGACAAACCGGAGGAAGGTGGGGAT

GACGTCAAATCATCATGCCCCTTATGATTTGGGCTACACACGTGCTACAATGGATAATACAAA

GGGCAGCGAACCCGCGAGGTCAAGCAAATCCCATAAAATTATTCTCAGTTCGGATTGTAGTC

TGCAACTCGACTACATGAAGCTGGAATCGCTAGTAATCGTAGATCAGCATGCTACGGTGAATA

CGTTCCCGGGTCTTGTACACACCGCCCGTCACACCACGAGAGTTTGTAACACCCGAAGCCG

GTGGAGTAACCTTTTATTAGGAGCTAGCCGTCGAAAGGTGGAACAAGAATGT

>NA4 *Escherichia*

ATAAATTGCCGGGCAGCCTTAACACATGCAAGTCGAACGGTAACAGGAAGCAAGCTTGCTG

CTTCGCTGACGAGTGGCGGACGGGTGAGTAATGTCTGGGAAACTGCCTGATGGAGGGGGAT

AACTACTGGAAACGGTAGCTAATACCGCATAACGTCGCAAGACCAAAGAGGGGGACCTTCG

GGCCTCTTGCCATCGGATGTGCCCAGATGGGATTAGCTAGTAGGTGGGGTAACGGCTCACCT

AGGCGACGATCCCTAGCTGGTCTGAGAGGATGACCAGCCACACTGGAACTGAGACACGGTC

CAGACTCCTACGGGAGGCAGCAGTGGGGAATATTGCACAATGGGCGCAAGCCTGATGCAGC

CATGCCGCGTGTATGAAGAAGGCCTTCGGGTTGTAAAGTACTTTCAGCGGGGAGGAAGGGA

GTAAAGTTAATACCTTTGCTCATTGACGTTACCCGCAGAAGAAGCACCGGCTAACTCCGTGC
CAGCAGCCGCGGTAATACGGAGGGTGCAAGCGTTAATCGGAATTACTGGGCGTAAAGCGCA
CGCAGGCGGTTTGTTAAGTCAGATGTGAAATCCCCGGGCTCAACCTGGGAACTGCATCTGAT
ACTGGCAAGCTTGAGTCTCGTAGAGGGGGGTAGAATTCCAGGTGTAGCGGTGAAATGCGTA
GAGATCTGGAGGAATACCGGTGGCGAAGGCGGCCCCCTGGACGAAGACTGACGCTCAGGT
GCGAAAGCGTGGGGAGCAAACAGGATTAGATACCCTGGTAGTCCACGCCGTAAACGATGTC
GACTTGGAGGTTGTGCCCTTGAGGCGTGGCTTCCGGAGCTAACGCGTTAAGTCGACCGCCT
GGGGAGTACGGCCGCAAGGTTAAAACTCAAATGAATTGACGGGGGCCCGCACAAGCGGTG
GAGCATGTGGTTTAATTCGATGCAACGCGAAGAACCTTACCTGGTCTTGACATCCACGGAAG
TTTTCAGAGATGAGAATGTGCCTTCGGGAACCGTGAGACAGGTGCTGCATGGCTGTCGTCAG
CTCGTGTTGTGAAATGTTGGGTTAAGTCCCGCAACGAGCGCAACCCTTATCCTTTGTTGCCA
GCGGTCCGGCCGGGAACTCAAAGGAGACTGCCAGTGATAAACTGGAGGAAGGTGGGGATG
ACGTCAAGTCATCATGGCCCTTACGACCAGGGCTACACACGTGCTACAATGGCGCATACAAA
GAGAAGCGACCTCGCGAGAGCAAGCGGACCTCATAAAGTGCGTCGTAGTCCGGATTGGAGT
CTGCAACTCGACTCCATGAAGTCGGAATCGCTAGTAATCGTGGATCAGAATGCCACGGTGAA
TACGTTCCCGGGCCTTGTACACACCGCCCGTCACACCATGGGAGTGGGTTGCAAAAGAAGT
AGGTAGCTTAACCTTCGGGAGGGCGGCTAACCCACTTTTGATTTAAGGGG

>BHIA1 *Staphyloccocus*

GGGGACGTTGCGGGGGTGCTATTACATGCAAGTCGAGCGAACAGATGAGAAGCTTGCTTCT
CTGATGTTAGCGGCGGACGGGTGAGTAACACGTGGGTAACCTACCTATAAGACTGGGATAAC
TCCGGGAAACCGGGGCTAATACCGGATAATATATTGAACCGCATGGTTCAATGTTGAAAGAC
GGTTTCGGCTGTCACTTATAGATGGACCCGCGCCGTATTAGCTAGTTGGTAAGGTAACGGCTT
ACCAAGGCAACGATACGTAGCCGACCTGAGAGGGTGATCGGCCACACTGGAACTGAGACAC
GGTCCAGACTCCTACGGGAGGCAGCAGTAGGGAATCTTCCGCAATGGGCGAAAGCCTGACG
GAGCAACGCCGCGTGAGTGATGAAGGTCTTAGGATCGTAAAACTCTGTTGTTAGGGAAGAA
CAAATTTGTTAGTAACTGAACAAGTCTTGACGGTACCTAACCAGAAAGCCACGGCTAACTAC
GTGCCAGCAGCCGCGGTAATACGTAGGTGGCAAGCGTTATCCGGAATTATTGGGCGTAAAGC

GCGCGTAGGCGGTTTCTTAAGTCTGATGTGAAAGCCCACGGCTCAACCGTGGAGGGTCATTG
GAAACTGGGGAACTTGAGTGCAGAAGAGGAGAGTGGAATTCCATGTGTAGCGGTGAAATGC
GCAGAGATATGGAGGAACACCAGTGGCGAAGGCGGCTCTCTGGTCTGTAACTGACGCTGAT
GTGCGAAAGCGTGGGGATCAAACAGGATTAGATACCCTGGTAGTCCACGCCGTAAACGATG
AGTGCTAAGTGTTAGGGGGTTTCCGCCCCTTAGTGCTGCAGCTAACGCATTAAGCACTCCGC
CTGGGGAGTACGACCGCAAGGTTGAAACTCAAAGGAATTGACGGGGACCCGCACAAGCGG
TGGAGCATGTGGTTTAATTCGAAGCAACGCGAAGAACCTTACCAAATCTTGACATCCTTTGA
TCGCTCTAGAGATAGAGTTTTCCCCTTCGGGGGACAAAGTGACAGGTGGTGCATGGTTGTCG
TCAGCTCGTGTCGTGAGATGTTGGGTTAAGTCCCGCAACGAGCGCAACCCTTAAGCTTAGTT
GCCATCATTAAGTTGGGCACTCTAGGTTGACTGCCGGTGACAAACCGGAGGAAGGTGGGGA
TGACGTCAAATCATCATGCCCCTTATGATTTGGGCTACACACGTGCTACAATGGATAATACAA
AGGGCAGCGAACCCGCGAGGTCAAGCAAATCCCATAAAATTATTCTCAGTTCGGATTGTAGT
CTGCAACTCGACTACATGAAGCTGGAATCGCTAGTAATCGTAGATCAGCATGCTACGGTGAAT
ACGTTCCCGGGTCTTGTACACACCGCCCGTCACACCGGCGAGAGTTTGTAACACCCGAAGC
CGGTGGAGTAACCTTTTATTAGGAGCTAGCCGTCGAAAGTTGAACCAGGATGT

>BHIA2 *Staphyloccocus*

AGAAACGTGCGGCGTGCTAATAATGCAAGTCGAGCGAACAGATAAGGAGCTTGCTCCTTTG
ACGTTAGCGGCGGAAGGGTGAGTAACACGTGGGTAACCTACCTATAAGACTGGGATAACTTC
GGGAAACCGGAGCTAATACCGGATAACATTTGGAACCGCATGGTTCTAAAGTGAAAGATGGT
TTTGCTATCACTTATAGATGGACCCGCGCCGTATTAGCTAGTTGGTAAGGTAACGGCTTACCA
AGGCGACGATACGTAGCCGACCTGAGAGGGTGATCGGCCACACTGGAACTGAGACACGGTC
CAGACTCCTACGGGAGGCAGCAGTAGGGAATCTTCCGCAATGGGCGAAAGCCTGACGGAGC
AACGCCGCGTGAGTGATGAAGGGTTTCGGCTCGTAAAACTCTGTTATTAGGGAAGAACAAAT
GTGTAAGTAACTGTGCACATCTTGACGGTACCTAATCAGAAAGCCACGGCTAACTACGTGCC
AGCAGCCGCGGTAATACGTAGGTGGCAAGCGTTATCCGGAATTATTGGGCGTAAAGCGCGCG
TAGGCGGTTTCTTAAGTCTGATGTGAAAGCCCACGGCTCAACCGTGGAGGGTCATTGGAAAC
TGGGAAACTTGAGTGCAGAAGAGGAAAGTGGAATTCCATGTGTAGCGGTGAAATGCGCAGA

GATATGGAGGAACACCAGTGGCGAAGGCGACTTTCTGGTCTGTAACTGACGCTGATGTGCG
AAAGCGTGGGGATCAAACAGGATTAGATACCCTGGTAGTCCACGCCGTAAACGATGAGTGCT
AAGTGTTAGGGGGTTTCCGCCCCTTAGTGCTGCAGCTAACGCATTAAGCACTCCGCCTGGGG
AGTACGACCGCAAGGTTGAAACTCAAAGGAATTGACGGGGACCCGCACAAGCGGTGGAGC
ATGTGGTTTAATTCGAAGCAACGCGAAGAACCTTACCAAATCTTGACATCCTTTGAAAACTC
TAGAGATAGAGCCTTCCCCTTCGGGGGACAAAGTGACAGGTGGTGCATGGTTGTCGTCAGCT
CGTGTCGTGAGATGTTGGGTTAAGTCCCGCAACGAGCGCAACCCTTAAGCTTAGTTGCCATC
ATTAAGTTGGGCACTCTAAGTTGACTGCCGGTGACAAACCGGAGGAAGGTGGGGATGACGT
CAAATCATCATGCCCCTTATGATTTGGGCTACACACGTGCTACAATGGACAATACAAAGGGCA
GCTAAACCGCGAGGTCATGCAAATCCCATAAAGTTGTTCTCAGTTCGGATTGTAGTCTGCAA
CTCGACTACATGAAGCTGGAATCGCTAGTAATCGTAGATCAGCATGCTACGGTGAATACGTTC
CCGGGTCTTGTACACACCGCCCGTCACACCACGAGAGTTTGTAACACCCGAAGCCGGTGGA
GTAACCATTTATGGAGCTAGCCCGTCGAAAGGTTGAACCCAGTGGG

>BHIA3 *Kocuria*

GGCAAAGGGCGGGGTGCTTAAAATGCAAGTCGAACGCTGAAGCTTGGTGCTTGCACCGGGT
GGATGAGTGGCGAACGGGTGAGTAATACGTGAGTGACCTACCTTGGACTCTGGGATAAGCCT
GGGAAACTGGGTCTAATACCGGATATGACCAATGGGCGCATGCTCTGTTGGTGGAAAGGGTT
TGTACTGGTCTTTGATGGGCTCACGGCCTATCAGCTTGTTGGTGAGGTAATGGCTTACCAAGG
CGACGACGGGTAGCCGGCCTGAGAGGGTGACCGGCCACACTGGGACTGAGACACGGCCCA
GACTCCTACGGGAGGCAGCAGTGGGGAATATTGCACAATGGGCGCAAGCCTGATGCAGCGA
CGCCGCGTGAGGGATGACGGCCTTCGGGTTGTAAACCTCTTTCAGCAGGGAAGAAGCGAAA
GTGACGGTACCTGCAGAAGAAGCGCCGGCTAACTACGTGCCAGCAGCCGCGGTAATACGTA
GGGCGCAAGCGTTGTCCGGAATTATTGGGCGTAAAGAGCTCGTAGGCGGTTTGTCGCGTCTG
CTGTGAAAGCCCGGGGCTTAACCCCGGGTGTGCAGTGGGTACGGGCAGGCTAGAGTGCAGT
AGGGGAGACTGGAATTCCTGGTGTAGCGGTGAAATGCGCAGATATCAGGAGGAACACCGAT
GGCGAAGGCAGGTCTCTGGGCTGTTACTGACGCTGAGGAGCGAAAGCATGGGGAGCGAAC
AGGATTAGATACCCTGGTAGTCCATGCCGTAAACGTTGGGCACTAGGTGTGGGGGACATTCC

ACGTTTTCCGCGCCGTAGCTAACGCATTAAGTGCCCCGCCTGGGGAGTACGGCCGCAAGGCT
AAAACTCAAAGGAATTGACGGGGGCCCGCACAAGCGGCGGAGCATGCGGATTAATTCGATG
CAACGCGAAGAACCTTACCAAGGCTTGACATACACCGGATCGCAGCAGAGATGTTGTTTCCT
TTCGGGGCTGGTGTACAGGTGGTGCATGGTTGTCGTCAGCTCGTGTCGTGAGATGTTGGGTT
AAGTCCCGCAACGAGCGCAACCCTCGTTCTATGTTGCCAGCACGTGATGGTGGGGACTCATA
GGAGACTGCCGGGGTCAACTCGGAGGAAGGTGGGGATGACGTCAAATCATCATGCCCCTTA
TGTCTTGGGCTTCACGCATGCTACAATGGCCGGTACAATGGGTTGCGATACTGTGAGGTGGA
GCTAATCCCAAAAAGCCGGTCTCAGTTCGGATTGGGGTCTGCAACTCGACCCCATGAAGTCG
GAGTCGCTAGTAATCGCAGATCAGCAACGCTGCGGTGAATACGTTCCCGGGCCTTGTACACA
CCGCCCGTCAAGTCACGAAAGTCGGTAACACCCGAAGCCGATGGCCTAACCCTTTGTGGGG
GGAGTCGTCGAAGGGTTGAGCCGTTAAAC
